# Genomics and transcriptomics yields a system-level view of the biology of the pathogen *Naegleria fowleri*

**DOI:** 10.1186/s12915-021-01078-1

**Published:** 2021-07-22

**Authors:** Emily K. Herman, Alex Greninger, Mark van der Giezen, Michael L. Ginger, Inmaculada Ramirez-Macias, Haylea C. Miller, Matthew J. Morgan, Anastasios D. Tsaousis, Katrina Velle, Romana Vargová, Kristína Záhonová, Sebastian Rodrigo Najle, Georgina MacIntyre, Norbert Muller, Mattias Wittwer, Denise C. Zysset-Burri, Marek Eliáš, Claudio H. Slamovits, Matthew T. Weirauch, Lillian Fritz-Laylin, Francine Marciano-Cabral, Geoffrey J. Puzon, Tom Walsh, Charles Chiu, Joel B. Dacks

**Affiliations:** 1 Division of Infectious Disease, Department of Medicine, Faculty of Medicine and Dentistry, University of Alberta, Edmonton, Canada; 2 Department of Agricultural, Food and Nutritional Science, University of Alberta, Edmonton, Alberta Canada; 3 Laboratory Medicine and Medicine / Infectious Diseases, UCSF-Abbott Viral Diagnostics and Discovery Center, UCSF Clinical Microbiology Laboratory UCSF School of Medicine, San Francisco, USA; 4 Department of Laboratory Medicine, University of Washington Medical Center, Montlake, USA; 5 Centre for Organelle Research, Department of Chemistry, Bioscience and Environmental Engineering, University of Stavanger, Stavanger, Norway; 6 School of Applied Sciences, Department of Biological and Geographical Sciences, University of Huddersfield, Huddersfield, UK; 7 Department of Cardiology, Hospital Clinico Universitario Virgen de la Arrixaca. Instituto Murciano de Investigación Biosanitaria. Centro de Investigación Biomedica en Red-Enfermedades Cardiovasculares (CIBERCV), Madrid, Spain; 8 CSIRO Land and Water, Centre for Environment and Life Sciences, Private Bag No.5, Wembley, Western Australia 6913 Australia; 9 CSIRO, Indian Oceans Marine Research Centre, Environomics Future Science Platform, Crawley, WA Australia; 10 CSIRO Land and Water, Black Mountain Laboratories, Canberra, Australia; 11 School of Biosciences, University of Kent, Canterbury, UK; 12 Department of Biology, University of Massachusetts, Amherst, UK; 13 Department of Biology and Ecology, Faculty of Science, University of Ostrava, Ostrava, Czech Republic; 14 Faculty of Science, Charles University, BIOCEV, Prague, Czech Republic; 15 Institute of Parasitology, Biology Centre, Czech Academy of Sciences, České Budějovice, Czech Republic; 16 Institut de Biologia Evolutiva (UPF-CSIC), Barcelona, Spain; 17 Centre for Genomic Regulation (CRG), Barcelona Institute of Science and Technology (BIST), 08003 Barcelona, Catalonia Spain; 18 Department of Medicine, Faculty of Medicine and Dentistry, University of Alberta, Edmonton, Canada; 19 Institute of Parasitology, Vetsuisse Faculty Bern, University of Bern, Bern, Switzerland; 20 Spiez Laboratory, Federal Office for Civil Protection, Austrasse, Spiez, Switzerland; 21 Department of Ophthalmology, Inselspital, Bern University Hospital, University of Bern, Bern, Switzerland; 22 Department of Biochemistry and Molecular Biology, Centre for Comparative Genomics and Evolutionary Bioinformatics, Dalhousie University, Halifax, Canada; 23 Center for Autoimmune Genomics and Etiology and Divisions of Biomedical Informatics and Developmental Biology, Cincinnati Children’s Hospital Medical Center, Cincinnati, OH USA; 24 Department of Pediatrics, University of Cincinnati College of Medicine, Cincinnati, USA; 25 Department of Microbiology and Immunology, Virginia Commonwealth University School of Medicine, Richmond, Virginia USA; 26 Department of Life Sciences, The Natural History Museum, London, UK

**Keywords:** Illumina, RNA-Seq, Genome sequence, Protease, Cytoskeleton, Metabolism, Lysosomal, Inter-strain diversity, Neuropathogenic

## Abstract

**Background:**

The opportunistic pathogen *Naegleria fowleri* establishes infection in the human brain, killing almost invariably within 2 weeks. The amoeba performs piece-meal ingestion, or trogocytosis, of brain material causing direct tissue damage and massive inflammation. The cellular basis distinguishing *N. fowleri* from other *Naegleria* species, which are all non-pathogenic, is not known. Yet, with the geographic range of *N. fowleri* advancing, potentially due to climate change, understanding how this pathogen invades and kills is both important and timely.

**Results:**

Here, we report an -omics approach to understanding *N. fowleri* biology and infection at the system level. We sequenced two new strains of *N. fowleri* and performed a transcriptomic analysis of low- versus high-pathogenicity *N. fowleri* cultured in a mouse infection model. Comparative analysis provides an in-depth assessment of encoded protein complement between strains, finding high conservation. Molecular evolutionary analyses of multiple diverse cellular systems demonstrate that the *N. fowleri* genome encodes a similarly complete cellular repertoire to that found in free-living *N. gruberi*. From transcriptomics, neither stress responses nor traits conferred from lateral gene transfer are suggested as critical for pathogenicity. By contrast, cellular systems such as proteases, lysosomal machinery, and motility, together with metabolic reprogramming and novel *N. fowleri* proteins, are all implicated in facilitating pathogenicity within the host. Upregulation in mouse-passaged *N. fowleri* of genes associated with glutamate metabolism and ammonia transport suggests adaptation to available carbon sources in the central nervous system.

**Conclusions:**

In-depth analysis of *Naegleria* genomes and transcriptomes provides a model of cellular systems involved in opportunistic pathogenicity, uncovering new angles to understanding the biology of a rare but highly fatal pathogen.

**Supplementary Information:**

The online version contains supplementary material available at 10.1186/s12915-021-01078-1.

## Background

**Fig. 1:**
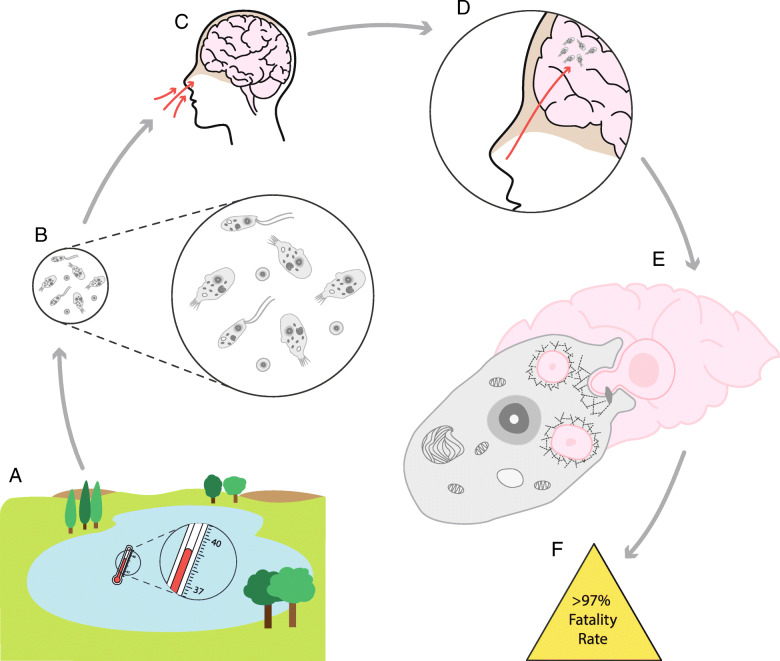
Infection of humans by *Naegleria fowleri*. (**a**) *N. fowleri* is found in warm fresh waters, (**b**) living primarily as an amoeboid trophozoite but also showing flagellate and cyst forms. If water containing *N. fowleri* enters the human nose (**c**), the trophozoite can opportunistically infect. (**d**) After passing through the cribriform plate (light-brown) (**e**), the amoeba phagocytoses brain material in a process of piece-meal ingestion called trogocytosis. (**f**) While treatable by a therapeutic cocktail if detected early, *N. fowleri* infection has an ~ 97% death rate

*Naegleria fowleri* is an opportunistic pathogen of humans and animals (Fig. 1), causing primary amoebic meningoencephalitis, and killing up to 97% of those infected, usually within 2 weeks [1]. It is found in warm freshwaters around the world and drinking water distribution systems [2–4] with *N. fowleri*-colonized drinking water distribution systems linked to deaths in Pakistan [5–7], Australia [8], and the USA [9, 10]. Human infection occurs when contaminated water enters the nose (Fig. 1). Opportunistically, *N. fowleri* passes through the cribriform plate to the olfactory bulb in the brain and performs trogocytosis (i.e., piece-meal ingestion) of brain material (Fig. 1), causing physical damage and massive inflammation leading to death. Although successful treatment with miltefosine and other antimicrobials is becoming more common [11, 12], this relies on appropriate early diagnosis, which is challenging due to the comparatively low incidence of amoebic versus viral or bacterial meningitis.

The known incidence of infection is relatively low, with 381 human cases reported in the literature [13]. However, infection is likely more prevalent. It was recently modelled that there are ~ 16 undetected cases annually in the US alone [14] and this situation could well be more pronounced in developing countries with warm climates and inconsistent medical reporting. *N. fowleri* has been recently proposed as an emerging pathogen, based on increased case reports in the past decade [15] and an increase in its northward expansion in the USA [16]. With cases from temperate locations reported in recent years [17, 18], the threat of *N. fowleri* may be exacerbated by range expansion due to climate change [15], which has been associated with a rise in freshwater temperatures, an increase in aquatic recreational activities [19], and extreme weather events. It is likely that we are still not fully aware of the global scale of *N. fowleri* infection [15], or how infection may increase as climate change accelerates.

Of the ~ 47 species of *Naegleria*, found commonly in soils and fresh waters worldwide, *N. fowleri* is the only species that infects humans, suggesting that pathogenicity is a gained function [20]. Several labs have identified potential *N. fowleri* pathogenicity factors including proteases, lipases, and pore-forming proteins [21–26]. The hypothesized mechanism of *N. fowleri* pathogenicity—tissue degradation for both motility during infection and phagocytosis—is compatible with these factors. However, none appear to be unique to *N. fowleri*, and there are likely proteins yet to be identified that are responsible for pathogenesis. In 2010, the genome of the non-pathogenic *N. gruberi* was published [27], followed by draft genomes of *N. fowleri* [28, 29]. This has set the stage for a thorough in-depth comparative genome-wide perspective on *N. fowleri* diversity and pathogenesis, which is currently lacking.

Here, we report the genome sequences of two *N. fowleri* strains; 986, an environmental isolate from an operational drinking water distribution systems in Western Australia, and CDC:V212, a strain isolated from a patient. We also report a transcriptomic analysis of induced pathogenicity in a third strain of *N. fowleri* (LEE) to identify the genes differentially expressed as a consequence of infection. Guided by these data, our careful curated comparative analysis of cellular machinery provides a comprehensive view of the pathogen *N. fowleri* at a cellular systems level.

## Results

### Two new *N. fowleri* genomes

In order to better understand the cellular basis for *N. fowleri* pathogenesis, we took a combined genomic, transcriptomic, and molecular evolutionary approach. The genomes of *N. fowleri* strains V212 and 986 were sequenced to average coverage of 251X and 250X respectively and the transcriptome of axenically cultured *N. fowleri* V212 was also sequenced to support gene prediction. Assembly statistics for our new genomes, as well as of the *N. fowleri* strain 30863, are shown in Table 1. Comparison between the three strains (Table 2) showed relative conservation of genome statistics. However, these were remarkably different from those values for *N. gruberi* (Table 2) [27]. The differences in genome statistics are not unreasonable given that genetic diversity within the *Naegleria* clade has been equated to that of tetrapods, at ~ 95% identity in the 18S rRNA gene ([30], Additional File 1-Table S1). Importantly, there is transcriptomic evidence for 82% of genes in *N. fowleri* V212, suggesting that they are expressed when grown in culture and 100% of the genes with transcriptome evidence were also found in predicted genomic set, meaning that we did not detect any transcribed genes that were not predicted from the genome. Out of 303 near-universal single-copy eukaryotic orthologs, 277 were found in the set of *N. fowleri* V212 predicted proteins, giving a BUSCO score of 88.3%, signifying that the genome and predicted proteome are highly complete.

**Table 1:** Assembly statistics for *N. fowleri* strains V212, 986, and ATCC 30863

	***N. fowleri*** V212	***N. fowleri*** 986	***N. fowleri*** 30863
Number of scaffolds	1859	990	1124
Total size of scaffolds	27,711,821	27,495,188	29,619,856
Longest scaffold	387,133	390,775	471,424
Mean scaffold size	14,907	2354	26,352
N50	92,316	101,682	136,406
L50	86	83	63
Number of contigs	1962	1919	2530
Total size of contigs	27,703,916	27,397,881	28,636,847
Longest contig	372,317	272,583	236,403
Mean contig size	14,120	14,277	11,319
N50	86,051	45,674	38,800
L50	93	182	213
BUSCO complete	88.3%	87.9%	87.8%
BUSCO single copy	76.9%	76.5%	73.7%
BUSCO duplicate	11.4%	11.4%	14.1%
BUSCO fragmented	2.4%	3.1%	2.7%
BUSCO missing	9.3%	9.0%	9.5%

**Table 2:** Genome statistics for *N. fowleri* strains V212, 986, 30863, and *N. gruberi* strain NEG-M

	***N. fowleri V212***	***N. fowleri 986***	***N. fowleri ATCC 30863***	***N. gruberi NEG-M***
Total genome size	27.7 Mbp	27.5 Mbp	29.62 Mbp	41.0 Mbp
GC content	36%	36%	35%	33%
Number of genes	12,677	11,599	11,499	15,708
Average gene length	1785 bp	1955 bp	1984 bp	1677 bp
Exons/gene	2	2	2	1.7
Average exon length	777 bp	849 bp	825 bp	894 bp
% coding	71.35%	73.01%	70.79%	57.80%
Average intron length	126 bp	138 bp	144 bp	203 bp

### *Naegleria fowleri* encodes a complete and canonical cellular complement

In 2010, *N. gruberi* was hailed as possessing extensive eukaryotic cytoskeletal, membrane trafficking, signaling, and metabolic machinery, suggesting sophisticated cell biology for a free-living protist that diverged from other eukaryotic lineages over one billion years ago [27, 31]. Careful manual curation of gene models and genomic analysis, as detailed in the “Methods” and Supplementary Material, of meiotic machinery (Additional File 2-Table S2, Additional File 3-Figure S1), transcription factors (Additional File 4-Supplementary Material 1, Additional File 5-Table S3, [32–35]), sterols (Additional Material 3-Figures S2 and S3, Additional File 4-Supplementary Material 2, Additional File 6-Table S4, [36–46]), mitochondrial proteins (Additional File 7-Table S5), cytoskeletal proteins (Additional File 3-Figure S4, Additional File 4-Supplementary Material 3, Additional File 8-Table S6, [21, 24, 27, 28, 47–64]), membrane trafficking components (Additional File 9-Table S7), and small GTPases (Additional File 3-Figures S5, S6, and S7, Additional File 4-Supplementary Material 4, Additional File 10-Table S8, [27, 65–79]) demonstrates that, like *N. gruberi*, *N. fowleri* possesses a remarkably complete repertoire of cellular machinery.

### Comparative genomics identifies hundreds of genes unique to *N. fowleri*

**Fig. 2:**
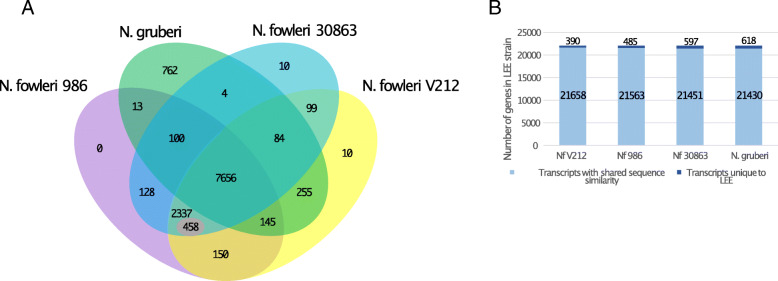
Genome and transcriptome conservation across *Naegleria* species. **a** Result of OrthoMCL analysis showing the number of orthogroups shared between the three *N. fowleri* strains and *N. gruberi*. The number of in-paralogue groups within each species is also shown (whereas strain-specific singletons are omitted from the diagram). The value 458 shown within the intersection of the three *N. fowleri* strains to the exclusion of *N. gruberi* is the number of orthogroups that did not retrieve any clear homolog in *N. gruberi* in a manual BLAST search. **b** Transcripts in the *N. fowleri* LEE transcriptome that share sequence similarity with genes in other *Naegleria* genomes based on BLAST analysis. Sequences with shared similarity are not considered to be necessarily orthologous

Pathogenesis is likely a gain-of-function. As *N. fowleri* is the only human pathogenic *Naegleria*, we specifically looked for differences with the non-pathogenic *N. gruberi*. Using OrthoMCL, the proteins from the three *N. fowleri* strains and *N. gruberi* were clustered into 11,399 orthogroups, of which 7656 (67%) appear to be shared by all four *Naegleria* species, and 10,451 (92%) are shared by all three *N. fowleri* strains (Fig. 2a). There are 2795 groups not identified in *N. gruberi* that are shared by all three *N. fowleri* strains. This number can be further split into orthogroups where BLAST searches against either the *N. gruberi* predicted proteome or the genome retrieve a potential homolog (these may be paralogs, or false negatives from OrthoMCL analysis), and groups that have no hit, and therefore no homolog, in *N. gruberi*. There are 458 orthogroups in *N. fowleri* whose members retrieve no *N. gruberi* homologs (Additional File 11-Table S9). Of these, 80% are unique to *N. fowleri*, with no clearly homologous sequence in any other organisms based on NCBI BLAST, and only 52 (11%) identified either could be functionally annotated based on NR BLAST results or contain a characterized domain. In total, 404 of the 458 genes have transcriptomic evidence (FPKM > 5) in either the axenic or mouse-passaged sample groups, suggesting that most of the gene models are accurate and these genes are expressed.

### Transcriptomics identifies differentially expressed genes in an animal model of *N. fowleri* pathogenesis

To complement the comparative genomics approach, we also performed transcriptomics, taking advantage of previously established experimentally induced pathogenicity in the *N. fowleri* LEE strain [80]. In this system, not only does the strain that has been continuously passaged in mice have a lower LD_50_ in guinea pigs by two orders of magnitude, it is also resistant to complement-mediated killing, while cultured *N. fowleri* LEE and *N. gruberi* are not [80]. De novo assembly of the *N. fowleri* LEE transcriptome shows that there are relatively few gene transcripts that do not share any similar sequence with other *N. fowleri* strains (Fig. 2b).

We identified 315 differentially expressed genes in mouse-passaged *N. fowleri* LEE (LEE-MP) compared to *N. fowleri* LEE grown in culture (LEE-Ax) (Additional File 12-Table S10). Of these, 206 are upregulated in mouse-passaged *N. fowleri*, while 109 are downregulated (Additional File 3- Figure S8). In terms of function, these 206 genes span multiple cellular systems with potential links to pathogenesis. Overall analysis of downregulated genes was less informative than that of upregulated genes. Systems represented in the downregulated gene dataset include signal transduction, flagellar motility, genes found to be involved in anaerobiosis in bacteria, and transcription/translation (Additional File 12-Table S10). Nonetheless, approximately 70% of the downregulated genes not in these categories are genes of unknown function.

Building on the manual curation of the encoded cellular machinery and using the comparative genomic and transcriptomic data, we confirmed and extended our knowledge of previously identified categories of pathogenicity factors and identified several novel major aspects of *N. fowleri* biology with implications for pathogenesis and that represent avenues for future investigation.

### Proteases and endolysosomal proteins are a substantial system in *N. fowleri*

Similar to many other animal parasites, *N. fowleri* is known to secrete proteases that traverse the extracellular matrix during infection, and pore-forming proteins that kill host cells [21–25]. Prosaposin is the most prominent pore-forming protein (termed Naegleriapore A) and is heavily glycosylated and protease-resistant [22]. The precursor proteins for Naegleriapore A and B were indeed found in our upregulated DE gene set. Similarly, the cathepsin protease (Cathepsin A, or Nf314) is upregulated in mouse-passaged *N. fowleri*, consistent with its previously proposed role in pathogenicity [81].

Comparative genomics found broadly similar complements of proteases between all *N. fowleri* strains and *N. gruberi* (Additional File 3-Figure S9, Additional File 13-Table S11), with one exception. The serine protease S81 was found in all three *N. fowleri* genomes, but not in *N. gruberi*, and is highly (but not differentially) expressed under both axenic and mouse-passaged conditions with FPKM values ranging from 500 to 800. S81 protein has invertebrate-type lysozyme (ilys) and peptidoglycan-binding domains. Sequence-similarity searches against the NCBI non-redundant and EukProt databases to look for homologs identified only two candidates, both from foraminiferans (Additional File 14-Table S12). Subsequent searches using one of these foraminiferan sequences identified further hits in both databases. However, the region of putative homology between S81 and any retrieved sequences was restricted to the ilys domain, with no similarity outside of the region. Ilys domains are found widely across eukaryotes (Additional File 14-Table S12). Therefore, while S81 likely arose from a common ilys of unknown origin, it does not have clear orthologs in other organisms and appears to be *N. fowleri*-specific.

Twenty-eight proteases are upregulated in mouse-passaged *N. fowleri*, making up more than 10% of all upregulated genes (Additional File 12-Table S10). Of the protease families with *N. fowleri* homologs upregulated following mouse passage, half are either localized to lysosomes or are secreted. The most substantially represented types of lysosomal/secreted protease in the upregulated genes are the cathepsin proteases; specifically, the C01 subfamily, with 10 out of 21 genes upregulated in mouse-passaged *N. fowleri*. The C01 subfamily of proteases includes cathepsins B, C, L, Z, and F. Each of these subfamilies has multiple members, up to 10 in the case of Cathepsin B, and members of the B, Z, and F subfamilies are upregulated.

Despite the large number of C01 subfamily cathepsin proteases in *N. fowleri* (20-21 members), *N. gruberi* encodes even more (35). Many of the *N. fowleri* and *N. gruberi* cathepsins have 1:1 orthology (Additional File 3-Figure S10), with at least three expansions that have occurred in the Cathepsin B clade in *N. gruberi*. These expansions account for most of the difference in paralog number between the two species, making up 12 of ~ 16 *N. gruberi*-specific C01 homologs. Notably, of the *N. fowleri* cathepsin genes that are upregulated in mouse-passaged *N. fowleri*, at least two (i.e., CatB7-N*fowleri*V212_g899.t2, CatB8-N*fowleri*V212_g10536.t1) lack orthologs in *N. gruberi*, raising the possibility of their specific involvement in pathogenesis.

Protease secretion is underpinned by the membrane trafficking system (the canonical secretion pathway) and autophagy-based unconventional secretion (for proteins lacking N-terminal signal peptides). In both cases, there are few differences in gene presence, absence, and paralog number in the different *Naegleria* genomes (Additional File 9-Table S7 and Additional File 15-Table S13). Strikingly, however, 42% of the upregulated genes are involved in lysosomal processes. In addition to the 22 proteases above, a lysosomal rRNA degradation gene is upregulated, as well as three subunits of the vacuolar ATPase proton pump (16, 21, and 116 kDa) responsible for acidification of both lysosomes and secretory vesicles. Endolysosomal trafficking genes Rab GTPase Rab32 (one of three paralogs) and the retromer component Vps35 were also upregulated.

### Proteins driving actin cytoskeletal rearrangements

While actin is known to drive many cellular processes in eukaryotes, Nf-Actin has been implicated in pathogenicity in *N. fowleri* due to its role in trogocytosis via food cup formation [56]. Furthermore, actin-binding proteins and upstream regulators of actin polymerization were reported to correlate with virulence [28]. While the complements of actin-associated machinery are similar between the *Naegleria* species, we notably identified a PTEN domain on one of the formin homologs in *N. fowleri* that was not identified in *N. gruberi* (Additional File 4-Supplementary Material 3). Since humans also do not encode formins of the PTEN family, if these PTEN formins are responsible for any vital *Naegleria* processes, they may represent useful drug targets.

Although *Naegleria* actin protein levels do not always correlate with transcript levels [27, 82], a single subunit of the Arp2/3 complex (Arp3) and the WASH complex member strumpellin were both upregulated in the mouse-passaged amoebae (Additional File 4-Supplementary Material 3, Additional File 8-Table S6, Additional File 12-Table S10). Similarly, we identified an upregulated RhoGAP22 gene and the serine/threonine protein kinase PAK3, which are involved in Rac1-induced cell migration in other species as well as an upregulated member of the gelsolin superfamily in the mouse-passaged *N. fowleri*, which may contribute to actin nucleation, capping, or depolymerization [83]. Although the shift from the environment in the mouse brain to tissue culture conditions prior to sequencing may have resulted in an upregulation in macropinocytosis, which can alter cell motility and the transcription of cytoskeletal genes in *Dictyostelium discoideum* [84, 85], the overall modulation of cytoskeletal protein encoding genes highlights the potential importance of cytoskeletal dynamics in promoting virulence.

### Neither LGT nor cell stress have substantially shaped *N. fowleri*’s unique biology

One obvious potential source of pathogenicity factors is lateral gene transfer of bacterial genes into *N. fowleri* to the exclusion of *N. gruberi* and other eukaryotes. However, of the 458 genes exclusive to *N. fowleri*, most are of unknown function (Additional File 11-Table S9) and of these, only 26 have Bacteria, Archaea, or viruses as the largest taxonomic group containing the top five BLAST hits. Furthermore, only one of the genes upregulated in mouse-passaged *N. fowleri* lacks a homolog in *N. gruberi*, and it has a potential homolog in *D. discoideum* and members of the Burkholderiales clade of bacteria (N*fowleri*V212_g4665, Additional File 12-Table S10).

Another potential reason for *N. fowleri*’s ability to infect humans and animals is the ability to survive the stresses of infection. However, we observed no obvious differences between the *N. fowleri* and *N. gruberi* complements of the ER-associated degradation machinery and unfolded protein response machinery that would suggest a differential ability to cope with cell stress (Additional File 16-Table S14). Furthermore, our transcriptomics analysis showed a general downregulation of cell stress systems, as well as DNA damage repair (Additional File 12-Table S10). This does not suggest that these systems are significantly involved in pathogenesis and our transcriptomics experiment reflects an organism that is not under duress.

### Beyond Nfa1, adhesion factors remain mysterious in *N. fowleri*

Since infection requires the ability to attach to cells of the nasal epithelium, differences between *N. fowleri* and *N. gruberi* in cell-cell adhesion factors may be relevant to pathogenesis. While we were unable to find evidence of a previously reported integrin-like protein [86] in any of the genomic data (based on sequence similarity to human integrin), we did find that another previously identified attachment protein, Nfa1, was highly expressed in both mouse-passaged and axenically cultured *N. fowleri* LEE (FPKM > 1000), providing further evidence supporting its previously reported role in pathogenicity [87]. It is likely that the integrin-like protein identified by Jamerson and colleagues is unrelated to animal integrins, although it appears to be recognized by antibodies to human β1 integrin.

*N. fowleri* and *N. gruberi* encode relatively few putative adhesion G protein-coupled receptors (AGPCRs), with 10 or fewer in each organism (Additional File 17-Table S15, Additional File 18-Table S16). These were identified by searching for proteins with the appropriate domain organization: sequences that have both an extracellular domain (assuming correctly predicted topology in the membrane) and seven transmembrane regions. A full collection of repeat-containing proteins in *N. fowleri* V212 and *N. gruberi* is shown in Additional File 17-Table S15. Of the proteins involved in adhesion in *D. discoideum* (TM9/Phg1, SadA, SibA, and SibC), only orthologs of the TM9 protein could be reliably identified (Additional File 19-Table S17), which was not found to be differentially regulated in our transcriptomic dataset. It is possible that the *Naegleria* homolog may be involved in cell adhesion, but with other downstream effectors.

### *N. fowleri* shows modulation and unusual metabolic pathways

Strikingly, 19% of upregulated genes are involved in metabolism (Additional File 12-Table S10). Both catabolic and anabolic processes are represented; some upregulated genes include phospholipase B-like genes necessary for beta-oxidation, and genes involved in phosphatidate/phosphatidylethanolamine, fatty acid (including long-chain fatty acid elongation), and isoprenoid biosynthesis. Phospholipase B was previously identified as pathogenicity factors in *N. fowleri* [26]*.* Also identified in our study was a Rieske cholesterol C7(8)-desaturase (Additional File 3-Figure S3, Additional File 4-Supplementary Material 2), a protein involved in sterol production that is absent in mammals, thus potentially representing a drug target.

Recent work shows that *N. gruberi* trophozoites prefer to oxidize fatty acids to generate acetyl-CoA, rather than use glucose and amino acids as growth substrates [88]. Several genes involved in metabolism of both lipids and carbohydrates are upregulated in mouse-passaged *N. fowleri.* Of interest are those that may be involved in metabolizing the polyunsaturated long-chain fatty acids that are abundant in the brain, such as long-chain fatty acyl-CoA synthetase and a delta 6 fatty acid (linoleoyl-CoA) desaturase-like protein. Consistent with possible shifting carbon source usage or increased growth rates, mitochondrial and energy conversion genes are upregulated, such as ubiquinone biosynthesis genes, isocitrate dehydrogenase (TCA cycle), complex I and complex III genes (oxidative phosphorylation), and a mitochondrial ADP/ATP translocase. Eight genes involved in amino acid metabolism are also upregulated.

**Fig. 3:**
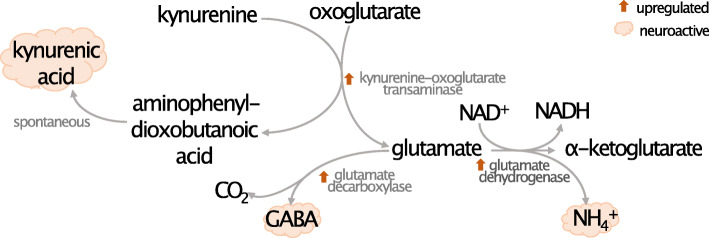
Mouse-passaged *N. fowleri* shows upregulated enzymes producing neuroactive chemicals. Upregulation of enzymes of glutamate metabolism in mouse-passaged LEE *N. fowleri* suggests a strategy for ATP production in vivo and synthesis of neuroactive metabolites

Intriguingly, we identified several areas of *N. fowleri* metabolism that may impact the human host. Glutamate is found in high millimolar concentrations in the brain and is thought to be the major excitatory neurotransmitter in the central nervous system [89, 90]. Several genes that function in glutamate metabolism are upregulated in mouse-passaged *N. fowleri*, including kynurenine-oxoglutarate transaminase, glutamate decarboxylase, glutamate dehydrogenase, and isocitrate dehydrogenase (Fig. 3, Additional File 12-Table S10). Multiple neurotropic compounds are generated via these enzymes, such as kynurenic acid, GABA, and NH_4_^+^. Kynurenic acid in particular has been linked to neuropathological conditions in tick-borne encephalitis [91].

Ammonia transporters are also upregulated (Additional File 12-Table S10), which may be one way to remove toxic ammonia from the cell, as *N. fowleri*, like *N. gruberi*, has an incomplete urea cycle [92]. Both glutamate and polyamine metabolism pathways discussed above generate NH_4_^+^ as a by-product, and it is possible that this is secreted into the host brain and leads to pathological effects.

Finally, agmatine deiminase, which is involved in putrescine biosynthesis, is also upregulated (Additional File 12-Table S10). This enzyme catalyzes the conversion of agmatine to carbamoyl putrescine, an upstream precursor to the polyamines glutathione and trypanothione [93]. Trypanothione provides a major defense against oxidative stress, some heavy metals, and potentially xenobiotics in trypanosomatid organisms (e.g., *Leishmania*, *Trypanosoma*) [94] and has been isolated from *N. fowleri* trophozoites [95]. Although a similar protective role for trypanothione has yet to be confirmed in *N. fowleri*, it is a critical component in trypanosomatid parasites [96, 97], and enzymes in this pathway may represent novel drug targets.

### Upregulated genes of miscellaneous or unknown function

There are many other genes that are upregulated, but do not fall into one of the categories outlined above (Additional File 12-Table S10). Notably, one of these is a transcription factor of the RWP-RK family, which has previously only been identified in plants and *D. discoideum*, functioning in plants to regulate responses in nitrogen availability, including differentiation and gametogenesis. While its role in amoebae is unclear, it may represent a potential drug target, as RWP-RK transcription factors are not present in human cells. Notably, of the 208 genes upregulated in highly pathogenic *N. fowleri*, 49 genes found either in *N. fowleri* alone or in *N. fowleri* and *N. gruberi* (Additional File 12-Table S10). These represent unique potential targets against which anti-*Naegleria* therapeutics may be developed.

## Discussion

In this study, we provide a comparative assessment of genomic encoded proteomes between *N. fowleri* strains and the first comprehensive system-level analysis to understand why this species of *Naegleria* is a highly fatal human pathogen while other species are essentially benign.

Our work is consistent with previous understanding of *N. fowleri* pathogenicity, but builds extensively upon it. Our transcriptomic analysis revealed increased expression of several genes previously considered as pathogenicity factors (e.g., actin, the prosaposin precursor gene of *Naegleria*pore A and B, phospholipases and Nf314 (Cathepsin A) [22, 56, 81]. In 2014, Zysset-Burri and colleagues published a proteomic screen of highly versus weakly virulent *N. fowleri*, as a function of culturing cells with different types of media [28]. While there were clear differences between these strains, potentially due to genetic differences and the method of virulence induction, there were some shared pathways. This included villin and severin, which were both more abundant in high virulence *N. fowleri* and are involved in actin cytoskeletal dynamics, as well as a phospholipase D homolog.

**Fig. 4:**
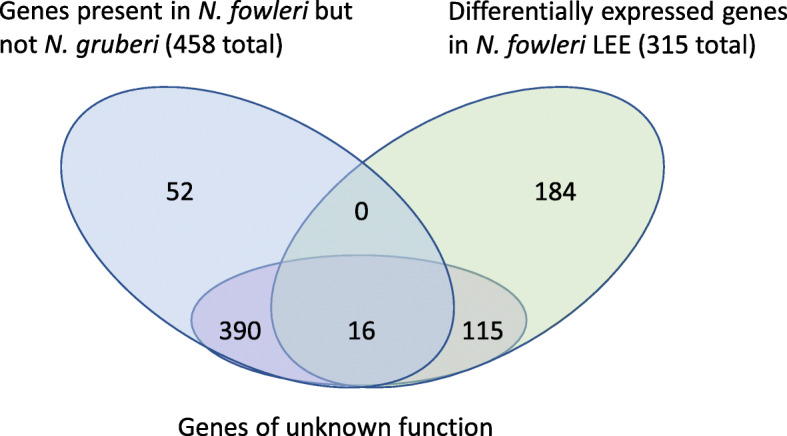
Venn diagram showing the overlap between differentially expressed genes in mouse-passaged *N. fowleri*, those which have no clear homologs in *N. gruberi*, and those of unknown function

However, our comparative genomics and transcriptomics approaches have identified many putative novel pathogenicity factors in *N. fowleri* (Fig. 4). We recognize that our comparison was limited to a single non-pathogenic species (*N. gruberi*) and so observed *N. fowleri*-specific aspects could be due to losses in *N. gruberi*. Given the more extensive predicted proteome of *N. gruberi* (15,708 genes) compared with the three *N. fowleri* strains considered here (average of 11,925 genes), we believe this effect to be minimal, but for any given protein the potential for this artifact exists. When multiple high-quality genomes from additional non-pathogenic *Naegleria* species become available, a re-analysis of the comparative genomic assessment will be worthwhile. Nonetheless, we identified key individual targets, such as the S81 protease and two cathepsin B proteases, which are both missing from *N. gruberi*, and differentially expressed in mouse-passaged *N. fowleri*. Moreover, taking a hierarchical approach of overlapping criteria, we can distill from high-throughput RNA-Seq data a catalog of novel potential pathogenicity factors. A total of 458 genes are shared by *N. fowleri* strains to the exclusion of *N. gruberi*, while 315 are differentially expressed upon pathogenicity inducing conditions; both are logical criteria for their consideration as potential pathogenicity factors. Annotation as “unknown function” was taken as a criterion for novelty, but not necessarily pathogenicity. At the intersection of these criteria, there are 390 genes of unknown function that are specific to *N. fowleri*, and 115 genes that are differentially expressed and are of unknown function. Sixteen genes fulfill all three criteria; they are specific to *N. fowleri*, are upregulated, and have no putative function. Notably, 90 of the upregulated genes do not appear to have a human ortholog and represent potential novel drug targets. Once genetic tools are developed in this lineage, these can be used to functionally characterize the most promising candidate genes and better study *N. fowleri* cell biology. These data would greatly improve our understanding of why and how it is so virulent.

**Fig. 5:**
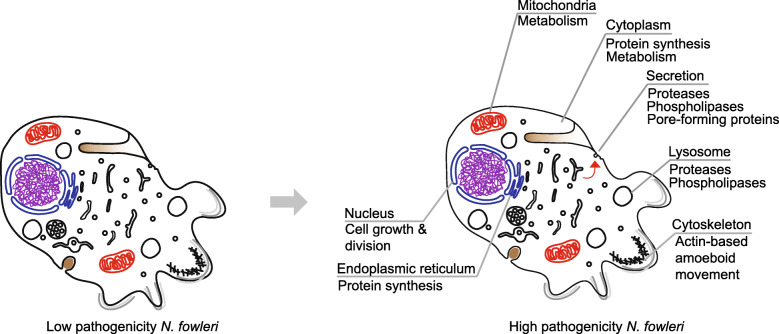
Model of *N. fowleri* pathogenicity. Aspects of cellular function that are likely relevant to *N. fowleri* pathogenicity are indicated on the cartoon of high-pathogenicity *N. fowleri* (right), as compared with low pathogenicity *N. fowleri* (left). This model does not represent an exhaustive list of all identified pathogenicity factors, but rather maps the system-level changes in *N. fowleri* based on the results of our differential gene expression analysis

Our work has allowed us to generate a model for pathogenicity in *N. fowleri* (Fig. 5), hinging on both specific protein factors as well as whole cellular systems. Secreted proteases (e.g., metalloproteases, cysteine proteases, and pore-forming proteases) and phospholipases involved in host tissue destruction are likely to be secreted by the cell’s membrane trafficking system. The lysosomal system clearly serves a major role, potentially both as a secretory route and in the degradation pathway, and is a major source of differentially expressed genes in our analysis. We identified many upregulated cathepsin proteases, which function in the lysosome, and predict that they are involved in ingestion and breakdown of host material. Increased cellular ingestion goes hand-in-hand with cell growth and division, processes which we also see represented in the upregulated gene dataset: genes involved in protein synthesis, metabolism, and mitochondrial function. This includes several metabolic pathways that produce compounds that could interact with the host immune system or have neurotropic effects. Finally, a major part of *N. fowleri*’s pathogenesis undoubtedly involves cell motility and phagocytosis, which are almost always actin-mediated processes.

## Conclusions

Overall, our comparative molecular evolutionary analysis, and the high-quality curated set of resources that have resulted, provides a rich and detailed cellular understanding of this enigmatic but unquestionably deadly human pathogen.

## Methods

### Culturing

The Australian *Naegleria fowleri* isolate 986 was obtained from an operational drinking water distribution system in rural Western Australia and verified as *N. fowleri* using qPCR-melt curve analysis [3] and DNA sequencing to confirm as Type 5 variant [98]. The Australian *N. fowleri* type 5 was cultured on axenic media, modified Nelson’s medium consisting of 1 g/L Oxoid Liver Digest (Oxoid), 1 g/L Glucose, 24.0 g/L NaCl, 0.40 g/L MgSO4-7H2O, 0.402 g/L CaCl-2H2O, 14.2 g/L Na2HPO4, 13.60 g/L KH2PO4, 10 % v/v Hyclone Fetal Bovine Serum (Thermo Fisher), heat-inactivated, 0.5% v/v Vitamin Mix (0.05 g D- biotin (Sigma), 0.05 g Folic acid (Sigma), and 2.5 g Sodium hydroxide) and Gentomycin (100 μg/mL), and incubated at 37 °C in 75 cm^2^ tissue culture flasks (Iwaki, Japan). Total DNA was extracted from the cultures as previously described using the PowerSoil DNA Isolation kit (MO BIO Laboratories, USA) [2, 99–101]. DNA was quantified using a Qubit (Invitrogen, USA) and stored at − 80 °C for subsequent genome sequencing.

*N. fowleri* V212 was cultured as described in Herman et al. [102]. *N. fowleri* LEE was grown axenically in oxoid media. Three replicates of *N. fowleri* LEE were passaged continuously through 50 B6C3F1 male mice. After mouse sacrifice, amoebae were extracted and grown in axenic media for 1 week to clear the culture of human cells prior to mRNA extraction.

### Genome sequencing and assembly

*Naegleria fowleri* V212 DNA was prepared and sequenced as described in Herman et al. [102]. Mitochondrial reads were first removed from 112,479,620 paired-end 100 bp Illumina and 609,044 454 reads using bowtie2 [103] and the remaining reads were de novo assembled using SPAdes v3.1.1 [104] and scaffolded using SSPACE [105], resulting in an assembly of 987 scaffolds > 200 bp totaling 27.7 Mb with an N50/N90 of 92,461/25,477 bp and depth of coverage of 251×. Notably, there are 366 sequences > 200 bp and < 1000 bp, comprising 387,133 bp or 1.4% of the assembly. Using the Geneious read mapper (22543367), 99.5% of 454 reads and 99.7% of Illumina reads mapped to the V212 mitochondrial genome and assembly.

For *N. fowleri* 986, a genomic sequencing library was constructed using the Illumina Nextera sequencing library kit and sequenced on an Illumina Hiseq 2000 (100 bp paired-end sequencing). Raw sequence reads were imported into CLC Genomics v 10.0.1 (Qiagen), quality trimmed using the default parameters and assembled using the denovo assembly program in CLC (minimum contig length 1000 bp). Using BWA-MEM [106], 99.61% of the reads from 986 matched to the 986 genome. Assembly statistics for V212, 986, and 30863 are provided in Table 1.

In Zysset-Burri [28], the ~ 30 Mb genome assembly of 30863 was taken to be a haploid assembly of the *N. fowleri* diploid genome based in a comparison to obtained flow cytometry data. Given the similarity in size of our assembled V212 and 986 genomes to that of 30863, we assume the same for these strains.

### Transcriptomics

For gene prediction purposes, mRNA was extracted from *N. fowleri* CDC:V212 grown in axenic culture. Sequencing was done using an Illumina HiSeq platform, and ~ 150 million paired-end reads were generated. These were quality filtered using Trimmomatic [107] and transcripts were de novo assembled using Trinity software [108] with default parameters. These transcripts were then used as hints to generate gene models using Augustus, as described below.

For differential expression of genes associated with pathogenicity in *N. fowleri*, mRNA was extracted from three independent cultures of *N. fowleri* LEE-MP (mouse passaged), and from *N. fowleri* LEE-AX (grown only in axenic culture). One microgram of RNA was converted to first-strand cDNA in a 25 μl volume (USB M-MLV RT). Twenty microliters (from 25 μl total volume) from first-strand synthesis was converted to double-strand cDNA (dscDNA; NEBNext mRNA Second Strand Synthesis Module, #E6111S). Twenty microliters of dscDNA was then used for library generation (Illumina Nextera XT library preparation kit). Ampure beads were used for sample clean-up throughout. Libraries were sequenced on an Illumina MiSeq (2 × 300 cycles; 600 V3 sequencing kit). Illumina MiSeq sequencing was performed at The Applied Genomics Centre at the University of Alberta, generating paired-end 2 × 300 reads. Reads were pre-processed using Trimmomatic v0.32400 [107], by adaptor trimming, 5′ end trimming (15 bp), trimming of regions where the average Phred score was < 20, and removal of short reads (< 50 bp). Remaining read set quality and characteristics were visualized using FastQC v0.11.2.401 [109].

The *N. fowleri* LEE-AX and LEE-MP reads were mapped to the *N. fowleri* V212 genome using TopHat v2.0.10 [110], with minimum intron length set to 30 bp, based on an assessment of predicted genes. Transcripts were then generated using Cufflinks with the Reference Annotation Based Transcript option, using the predicted genes as a reference dataset [111, 112]. The reference transcripts were tiled with “faux-reads” to aid in the assembly, and these sequences were added to the final dataset containing the newly assembled transcripts. In order to obtain transcripts not represented by genes in the *N. fowleri* V212 genome, Trinity (release 2013-02-25) was used for purely de novo transcriptome assembly, using a genome-guided approach, with a --genome_guided_max_intron value of 5000 [113, 114]. Novel Trinity-generated transcripts were added to the Cufflinks-generated transcriptome prior to downstream differential expression analyses. The reads were then re-assembled by Trinity de novo using default parameters with the exception of --jaccard_clip in order to ensure that no LEE-specific transcripts were missed.

To identify genes in the *N. fowleri* LEE transcriptome that do not have any sequence similarity with any predicted proteins in the other *N. fowleri* strains, the *N. fowleri* LEE transcripts were used as BLASTX queries to search the predicted proteomes of *N. fowleri* V212, 30863, and 986. In this analysis, sequences that retrieved any hit were considered to share sequence similarity with one or more genes in the other strains, and not necessarily directly orthologous.

### Differential expression analysis

Differential expression (DE) analyses were performed for the pathogenicity transcriptomic data using the programs Cuffdiff [114] and Trinity [113]. Cuffdiff was first used to map reads to the final transcriptome assembly, with high-abundance (> 10,000 reads) mitochondrial and extrachromosomal plasmid genes masked, and differential expression was calculated using geometric normalization. The Trinity Perl-to-R (PtR) toolkit was used to assess variation between replicates. One mouse passage replicate, MP2, was highly dissimilar to other mouse-passaged and axenically grown samples and was therefore excluded from further analysis. Using the Trinity suite of scripts, reads were aligned and transcript abundance estimated using RSEM [115] with TMM library normalization. Differentially expressed transcripts were then identified using EdgeR [116]. Comparison of these transcripts with those identified by Cuffdiff revealed highly overlapping datasets, with only a few genes considered to be differentially expressed by EdgeR but not Cuffdiff. Because this work is exploratory and we sought to minimize the potential for false negatives, we chose a lax false discovery rate cutoff of 0.1. However, the majority of upregulated genes have FDR values less than 0.05.

### Gene prediction and annotation

Gene prediction was performed using the program Augustus v.2.5.5 [117, 118] incorporating the HiSeq-generated transcript dataset as extrinsic evidence termed “hints”. Furthermore, Augustus was also trained using a manually annotated 60 kb segment from the *N. fowleri* V212 genome published previously [102]. This region was annotated by using it as a tBLASTn query to search the non-redundant database. Gene boundaries were identified using the alignments of the top hits, and genes were annotated based on top BLAST hit identities. Gene prediction was performed for the *N. fowleri* V212 and 986 genomes generated by this study, as well as on the publicly available genome for strain 30863. For each genome, the parameter --alternatives-from-evidence was set to true, as this reports alternative gene transcripts if there is evidence for them (i.e., from transcriptome dataset). The parameter --alternatives-from-sampling was also set to true, as this outputs additional suboptimal transcripts. Parameters for determining the importance of different hint data were kept as default.

Annotation of genes in specific subsystems of focus in this manuscript was done using sequence similarity searching to assess putative homology. Functionally characterized homologs of proteins of interest were used as BLASTP [119] queries to search the predicted proteins from the *N. fowleri* strain genomes. At a minimum, putative *N. fowleri* homologs must be retrieved with an E-value < 0.05. To be considered true homologs, the *N. fowleri* protein must retrieve the original query or a differently named version thereof in a reciprocal BLAST search also with an E-value < 0.05.

Additionally, specific methods to assess the repertoire of several cellular systems were additionally used. For the cytoskeletal components of the three *N. fowleri* isolates, we first compared *N. fowleri* proteins to previously identified *N. gruberi* actin and microtubule associated proteins using BLAST [119] against the protein databases generated in Augustus for each of the three *N. fowleri* isolates, using default search parameters. The top *N. fowleri* hits identified by comparisons to *N. gruberi* were then compared using BLAST to the full *N. gruberi* protein library to establish the Reciprocal Best Hit which are included in Additional File 8-TableS6. We then searched for additional proteins not found in *N. gruberi* using human or *Dictyostelium discoidium* protein sequences obtained from PubMed and dictyBase (http://dictybase.org), respectively*.* To identify *N. fowleri* homologs, we again used BLAST with the default parameters except replacing the default scoring matrix with BLOSUM45. We further validated protein identities through hmmscan searches using a gathering threshold and Pfam domains on the HMMER website (https://www.ebi.ac.uk/Tools/hmmer/search/hmmscan).

Protein domains of *N. fowleri* S81 protease (N*fowleri*V212_g9810.t1) were predicted using InterProScan [120] implemented in the Geneious Prime v2020.2.3 software [121]. Full-length sequence and sequence with the Invertebrate-type lysozyme (Lysozyme_I; IPR008597) domain removed were used as queries in BLASTp [122] searches in NCBI non-redundant and EukProt [123] databases. This analysis was repeated using one of the two retrieved sequences *Ammonia* sp. (CAMPEP_0197058342) as a query and both aforementioned databases. The validity of the retrieved sequences was verified by reverse BLASTp searches conducted against the *N. fowleri* protein database.

Mitochondrial protein location for all *N. fowleri* strains and the *N. gruberi* published genome [27] was determined based on a pipeline that tested several features. All predicted proteins were assessed for the following: (1) the presence of an N-terminal extension relative to bacterial/cytosolic homologs which was predicted as mitochondrial by Mitoprot [124], TargetP [125], and WoLFPSORT [126] and (2) a BLAST hit that was most significant against known mitochondrial proteins (and not against the non-mitochondrial paralogs from the same species). Biochemically confirmed mitochondrial proteomes from the following species were used for these BLAST searches: *Arabidopsis thaliana* [127], *Chlamydomonas reinhartdtii* [128], *Homo sapiens* [129], *Mus musculus* [129], *Tetrahymena thermophila* [130], and *Saccharomyces cerevisiae* [131]. In addition, mitosomal and hydrogenosomal predicted proteomes from the following species were also used in BLAST searches: *Giardia intestinalis* [132], *Entamoeba histolytica* [133], and *Trichomonas vaginalis* [134]. All hits were subsequently assessed against the Pfam [135] and SwissProt [136] databases. Proteins were ranked according to number of positive hits (e.g., mitochondrial targeting signal predicted by all predictors would be + 3 points, a positive hit against confirmed mitochondrial proteomes: maximum + 5, and the mitosomes/hydrogenosomal proteomes: maximum + 3). All predictions were manually curated, compared with the previously [27] and the newly curated *N. gruberi* mitochondrial proteins and subsequently spurious predictions were removed. Proteins most similar to unidentified proteins despite having a predicted mitochondrial targeting signal were removed as well.

For analysis of Ras super family GTPases, genomic and predicted protein sequences of *N. fowleri* strain V212, 986 and 30863, as well as *N. gruberi* were analyzed. For comparative analyses, we also identified Ras superfamily genes from a set of reference, phylogenetically diverse species using data available in the NCBI database (http://www.ncbi.nlm.nih.gov). Ras superfamily gene sequences were detected and preliminarily identified using the program BLAST and its variants [122]. The protein sequences were manually inspected and prediction of the underlying gene models corrected whenever necessary. Multiple protein alignments were constructed using the program MAFFT [137]. All alignments were checked and if necessary, further edited manually using BioEdit [138]. Phylogenetic trees were computed using the maximum likelihood method implemented in the program RAxML-HPC with the LG + Γ model and branch support assessed by the rapid bootstrapping algorithm [139] at the CIPRES Science Gateway portal [140]. The robustness was additionally assessed by the maximum likelihood method implemented in the IQ-tree program [141] with branch supports assessed by the ultrafast bootstrap approximation [142] and SH-aLRT test [143]. Genes with one-to-one orthology relationships were detected by maximum likelihood phylogenetic analysis (IQ-tree) or BLASTp searches. Two genes were considered as one-to-one orthologs if they form a monophyletic group to the exclusion of other analyzed sequences with support at least 80/95 (SH-aLRT support (%) / ultrafast bootstrap support (%)), or if they represent reciprocally best hits in BLASTp searches in our in-house Ras superfamily GTPase database containing also Ras superfamily genes from various other taxa. For the analysis of Rab proteins, the dataset from Elias et al. [72] was used and expanded with sequences from *Naegleria* spp. Protein domains were searched using SMART [144], Pfam [34], and NCBI’s conserved domain database [145]. Several suspicious domains detected only by NCBI’s conserved domain with low e-value and/or representing only a small part of detected domain were excluded from the results.

### Phylogenetic analysis

Bayesian and maximum likelihood phylogenetic analyses were performed to assign orthology to proteins from highly paralogous families. Sequences were aligned using MUSCLE v.3.8.31 [146], alignments were visualized in Mesquite v.3.2 [147], and manually masked and trimmed to remove positions of uncertain homology. ProtTest v3.4 [148] was used to determine the best-fit model of sequence evolution. PhyloBayes v4. 1[149] and MrBAYES v3.2.2 [150] programs were run for Bayesian analysis, and RAxML v8.1.3 [139] was run for maximum likelihood analysis. Phylobayes was run until the largest discrepancy observed across all bipartitions was less than 0.1 and at least 100 sampling points were achieved. MrBAYES was used to search treespace for a minimum of one million MCMC generations, sampling every 1000 generations, until the average standard deviation of the split frequencies of two independent runs (with two chains each) was less than 0.01. Consensus trees were generated using a burn-in value of 25%, well above the likelihood plateau in each case. RAxML was run with 100 pseudoreplicates.

### Genetic diversity analysis

As the highly cited analysis proposing that the genus *Naegleria* shows equivalent diversity to tetrapods [30] was based on only 281 nucleotides comparing four *Naegleria* species and five tetrapod sequences, we performed a more extensive analysis. For comparison purposes of *Naegleria* small subunit ribosomal RNA (18S rRNA) gene sequences, *Naegleria fowleri* 18S rRNA sequence (accession number NFT80059) was used as a query in BLASTN search [122] against nucleotide database on NCBI restricting search to the genus *Naegleria* and excluding *N. fowleri*. Only full-length sequences were used in further analysis. For comparison of tetrapods, 18S rRNA sequences were retrieved randomly with the aim to represent all major clades of tetrapods. 18S rRNA sequences were aligned using MAFFT v7.458 [137] under L-INS-i strategy. The resulting alignments were imported into the Geneious Prime v2020.2.3 software [121] and percent identities were obtained directly under “Distances” tab. Geometric mean and median were calculated using built-in functions in Excel. Overall, the hypothesis proposed by Baverstock [30] is supported but now with much more comprehensive data.

### BUSCO analysis

BUSCO v4.0.4 software [151, 152] was used to assess genome completeness. Predicted proteins were used as input, with the eukaryote dataset eukaryota_odb10 set of hidden Markov models.

### Orthologous groups analysis

To identify orthologous groups of sequences between the four *Naegleria* predicted proteomes, the program OrthoMCL v.2.0.9424 was used [153]. The Markov Clustering algorithm is an unsupervised clustering algorithm that clusters graphs based on pairwise scores (in this case, normalized E-values following an all-versus-all BLAST) and an inflation value. This latter value controls the clustering tightness and, for these analyses, was kept at the suggested 1.5.

For further manual analysis of potential orthogroups in *N. fowleri* strains but not *N. gruberi*, the above BLAST search criteria were used. Protein sequences from *N. fowleri* V212 were used to search the predicted proteome of *N. gruberi* using BLASTp, as well as the *N. gruberi* genome using tBLASTn. Any hits with an E-value less than 0.05 were used as queries in a reciprocal BLAST search against the *N. fowleri* V212 predicted proteome. *N. gruberi* sequences were considered homologous to the orthogroup protein if they retrieved the original query with an E-value < 0.05 (although the observed E-values were typically much lower). These relaxed BLAST criteria were designed to capture even highly divergent sequence, in order to be confident that the manually curated orthogroup dataset does not contain *N. gruberi* homologs. A total of 458 genes were identified as shared by all three *N. fowleri* strains and absent from *N. gruberi*.

### Transmembrane domain prediction

The program TMHMM v2.0426 [154, 155] was used to detect transmembrane helices in all *N. fowleri* V212 proteins. This software uses an HMM generated from 160 cross-validated membrane proteins, and outputs all predicted helices and protein orientation in the membrane. Because transmembrane helix prediction was done to generate a list of potential G protein-coupled receptors investigated further by domain prediction, scoring cutoffs were not used.

## Supplementary Information

**Additional file 1: Table S1.** % identity of SSU rRNA genes. Sheet 1: *Naegleria* species. Sheet 2: Selected tetrapod organisms.

**Additional file 2: Table S2.** Meiosis gene comparative genomics.

**Additional file 3.** **Figures S1-S10.**

**Additional file 4: Supplementary Material 1.** Transcription factor identification. **Supplementary Material 2.** Sterol metabolism genes from *Naegleria fowleri*. **Supplementary Material 3.** Analysis of the cytoskeletal protein complement in *N. fowleri*. **Supplementary Material 4.** Ras superfamily GTPases in *Naegleria* spp.

**Additional file 5: Table S3.** Transcription factors in *Naegleria* spp.

**Additional file 6: Table S4.** Genes involved in sterol production in *N. fowleri.*

**Additional file 7: Table S5.** Comparative genomic assessment of mitochondrial genes in *Naegleria* spp.

**Additional file 8: Table S6.** Cytoskeletal genes in *N. fowleri.*

**Additional file 9: Table S7.** Results of homology searching for membrane trafficking components in the predicted proteomes and genomes of *N. fowleri* V212, 39863, and 986.

**Additional file 10: Table S8.** Comparative genomics analysis of Ras superfamily GTPases in *Naegleria* spp.

**Additional file 11: Table S9.** Putative functional annotation of genes identified in *N. fowleri* but not *N. gruberi.*

**Additional file 12: Table S10.** Differentially expressed genes following mouse-passage of *N. fowleri* LEE. Grey shading indicates genes found in *N. fowleri* alone or *N. fowleri.*

**Additional file 13: Table S11.** Comparative genomic analysis of proteases identified in *Naegleria* spp.

**Additional file 14: Table S12.** Homology searching results for protease S81.

**Additional file 15: Table S13.** Comparative genomic analysis of genes involved in autophagy in *N. fowleri*.

**Additional file 16: Table S14.** Comparative genomic analysis of genes involved in the Unfolded Protein Response and the ER-Associated Degradation machinery in *N. fowleri.*

**Additional file 17: Table 15. **Repeat domain-containing proteins in *Naegleria* spp. identified by BLAST search using human adhesion G protein-coupled receptors as queries.

**Additional file 18: Table S16.** Predicted adhesion G protein-coupled receptors in *Naegleria* spp.

**Additional file 19: Table S17.** TM9 orthologues identified in *Naegleria* spp.

## Data Availability

Genomic sequence data for the three *N. fowleri* genomes (V212 [156], 986 [157], 30863 [28]) and the associated gff files are available on Zenodo https://zenodo.org/record/3951746#.XxW7xyhKhPZ [158]. Reads associated with the transcriptomics experiment of the LEE strain are deposited in the SRA (SRX8770894-SRX8770899) with the associated BioProject ID PRJNA647238 [159].
