# Supplementary material for: Genomics and transcriptomics yields a system-level view of the biology of the pathogen Naegleria fowleri
Source: BMC Biol. 2021 Jul 22;19:142. doi: 10.1186/s12915-021-01078-1 (PMC8296547; doi:10.1186/s12915-021-01078-1)
Supplement: Supplementary file 3 — Additional file 3. Figures S1-S10. [file 12915_2021_1078_MOESM3_ESM.pdf]

Figure Legends

Additional File 3-Figure S1. Dot plot showing the presence of meiosis genes in *Naegleria* sp. Meiosis gene in *N. gruberi* are based on Fritz-Laylin *et al.*, 2010. A filled circle indicates the presence of a gene, while an unfilled circle indicates that no clear homologue could be identified. Numbers within the circle indicate multiple paralogues. Like *N. gruberi*, *N. fowleri* encodes a highly complete complement of meiotic genes.

Additional File 3-Figure S2. Gene repertoire of the sterol synthesis pathway in *N. fowleri*. For comparison, the distribution of orthologues from S. cerevisiae, H. sapiens and A. thaliana, as well as the non-pathogenic N. gruberi, are shown. Double-dots indicate two orthologues performing one step (i.e. SMO1 and SMO2) or two orthologues performing similar reactions (i.e. SMT1 and SMT2). In the case of non-homologous families ERG2/EBP/HYD1 and ERG4/DHCR24/DWF1, the presence of one or the other copy is indicated by orange or blue dots as indicated in the gene names, whereas the presence of both copies is indicated by green dots. The Rieske cholesterol C7(8)-desaturase is shown apart because is not involved in the canonical sterol pathways.

Additional File 3-Figure S3. Partial alignment of cholesterol C7(8)-desaturases. Red boxes highlight the Rieske [2Fe-2S] motif (CXHX16CX2H) and the non-heme iron binding motif ((D/E)X3DX2HX4H). Asterisks indicate consensus residues important for catalysis.

Additional File 3-Figure S4. The *N. fowleri* genome encodes an extensive actin cytoskeletal repertoire. Formin family proteins typically nucleate and elongate actin filaments, moving progressively with the barbed end and recruiting profilin-bound actin monomers to the growing end of the filament. Another nucleator, the Arp2/3 complex, typically polymerizes a new filament from the side of a pre-existing filament following activation by a WASP-family protein such as WASP, SCAR/WAVE, and/or WASH. *N. fowleri* also encodes myosin motor proteins, including myosin I and II. Finally, *N. fowleri* encodes cofilin and members of the gelsolin/villin superfamily predicted to depolymerize actin filaments. In all, the *N. fowleri* genome encodes at least 22 actins, 14 formins, 11 myosins, 4 gelsolin/villin superfamily proteins, and 3 profilins, in addition to all the subunits of the Arp2/3 complex, and 3 WASP family proteins and their respective complexes.

Additional File 3-Figure S5. Phylogenetic analysis of Ras family genes in *Naegleria* species and other selected eukaryotes. Portrayed is a maximum likelihood tree (RAxML, LG+Γ model). Bootstrap support values were calculated using the rapid bootstrapping (Rboot) algorithm of the RAxML program. The robustness of the tree topology was also assessed by the IQ-tree with LG+F+G4 model (the model selected by the program itself) with the ultrafast bootstrap (UFboot) algorithm (1000 replicates) and the SH-aLRT test (1000 replicates). Circles at branches correspond to bootstrap values indicated in the legend. The bar on the top corresponds to the estimated number of substitutions per site. The identity of the *N. fowleri* genes is provided in Supplementary Table 7, sheet 2. Manually corrected gene models *; Newly created gene models**.

Additional File 3-Figure S6. Phylogenetic analysis of Rab family genes in *Naegleria* species and other eukaryotes from Elias et al., 2012 (Elias et al., 2012). Portrayed is a maximum likelihood tree (RAxML, LG+Γ model). Bootstrap support values were calculated using the rapid bootstrapping (Rboot) algorithm of the RAxML program. The robustness of the tree topology was assessed also by the IQ-tree with LG+G4 model (the model selected by the program itself) with

the ultrafast bootstrap (UFboot) algorithm (1000 replicates) and the SH-aLRT test (1000 replicates). Circles at branches correspond to bootstrap values indicated in the legend. The bar on the top corresponds to the estimated number of substitutions per site. The identity of the *N. fowleri* genes is provided in Supplementary Table 7, sheet 2. Manually corrected gene models *; Newly created gene models**.

Additional File 3-Figure S7. Multidomain proteins with a Ras superfamily GTPase domain found in Naegleria species. Selected proteins containing more than one domain are schematically depicted here. The positions of domains are according to NCBI's conserved domain database, except the C-terminal part of Gpa18 protein which was identified by manual inspection of the multiple sequence alignment of Naegleria spp. Gα proteins, as this fragment was too small to be detected by NCBI's conserved domain search. Ras superfamily domains are depicted in orange with a label specifying a subgroup of the Ras superfamily. ZnF UBP = Ubiquitin carboxyl-terminal hydrolase-like zinc finger; ANKs = multiple ankyrin repeats; G – alpha (Gα) = G protein alpha subunit; STKc = serine/threonine protein kinases. Nfo: *N. fowleri*; Ngr: *N. gruberi*; *: manually corrected gene model. The scale bar shows the length of 100 amino acid residues.

Additional File 3-Figure S8. MA plot and Volcano plot of genes differentially expressed in highly pathogenic *N. fowleri* LEE strain. MA plots show the log fold change of each gene relative to the log of the mapped read counts, while volcano plots scale the False Discovery Rate (FDR) to log fold change. Each gene is represented by a dot, and red dots indicate those which meet the differential expression criteria.

Additional File 3-Figure S9. Protease gene abundance in *Naegleria* per MEROPS protease family. For each MEROPS protease family, the number of predicted proteins in each strain is shown. The S81 family (boxed) is the only family without a homologue in *N. gruberi*. The only other known S81 family protease is a destabilase protein in *Hirudo medicinalis*, the European medicinal leech. Otherwise, most protease families could be identified in all four genomes, with similar numbers of homologues. The exception is the C01 cysteine protease family, which is analyzed in S8 Figure.

Additional File 3-Figure S10. Phylogenetic analysis of the C01 cysteine protease subfamily in *N. fowleri* and *N. gruberi*. Node values are listed as Phylobayes/RAxML (posterior probability/bootstrap), and as symbols indicating a minimum level of support as shown in the inset. Node values are shown on the best Bayesian topology. Sequences with signal peptides have red text, those with potential signal peptides (score near cutoff) have purple text, and those without identifiable signal peptides have blue text. Asterisks (*) indicate genes that are up-regulated in highly pathogenic *N. fowleri*.

Figure S1

	N. gruberi	*N. fowleri* V212	*N. fowleri* 30863	*N. fowleri* 986
Spo11*	❷	❷	❷	●
Mre11	❷	●	●	●
Rad50	●	●	●	●
Rad1	●	●	●	●
ERCC4	●	●	●	●
Hop1*	●	●	●	●
Hop2*	❷	❷	❷	❷
Mnd1*	●	●	●	●
Rad52	❸	❷	❷	❷
Dmc1*	●	●	●	●
Rad51	❸	❺	❺	❺
Msh2	●	●	❷	●
Msh6	❷	●	●	●
Msh3	●	●	●	●
Msh4*	●	●	●	●
Msh5*	●	●	●	●
Mlh1	●	●	●	●
Mlh2	○	○	○	○
Mlh3	●	●	●	●
Pms1/2	❷	❷	❷	❷
Mer3*	●	●	●	●
Smc1	●	●	●	●
Smc2	●	●	●	●
Smc3	●	●	●	●
Smc5	●	●	●	●
Rad18	●	●	●	●
Rad21	●	●	●	●
Rec8*	○	○	○	○
Pds5	●	●	●	●
Sec3	●	●	●	●

Based on Malik *et al.* 2008 and Fritz-Laylin *et al.* 2010
* Considered to be meiosis-specific in Malik *et al.* 2008

Figure S2

	S. cerevisiae	*H. sapiens*	*A. thaliana*	*N. gruberi*	***N. fowleri***
Squalene monooxygenase (ERG1/SQLE/SQE)	●	●	●	●	●
Oxidosqualene cyclase (ERG7/LSS/CAS1)	●	●	●	●	●
C-14 demethylase (ERG11/CYP51A1/CYP51G1)	●	●	●	●	●
C14-reductase (ERG24/TM7SF2/FK)	●	●	●	●	●
C-4 methyl oxydase (ERG25/SC4MOL/SMO1-2)	●	●	●●	●●	●●
C-3 dehydrogenase (ERG26/NSDHL/AT3betaHSD)	●	●	●	●	●
3-keto reductase (ERG27/HSD17B7/?)	●	●	○	○	○
ER anchor (ERG28/C14orf1/ERG28)	●	●	●	●	●
C-24 methyltransferase (ERG6/ - /SMT1-2)	●		●●	●	●
Δ8-Δ7 isomerase (ERG2/EBP/HYD1)	● (orange)	● (blue)	● (blue)	● (green)	● (green)
C-5 desaturase (ERG3/SC5DL/STE1)	●	●	●	●	●
Δ7(8)-reductase (- /DHCR7/DWF5)		●	●	●	●
C-22 desaturase (ERG5/ - /CYP710A1)	●		●		
Δ24-reductase (ERG4/DHCR24/DWF1)	● (orange)	● (blue)	● (blue)	● (orange)	● (orange)
Cyclopropyl isomerase (CPI1)			●	●	●
Cholesterol C7(8)-desaturase (Nvd/DAF36/Des7)				●	●

Figure S3

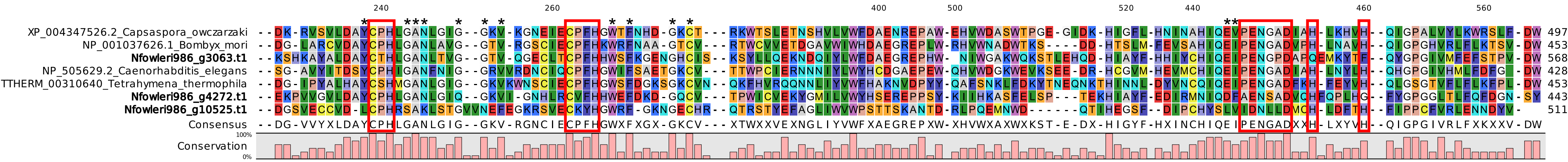
XP_004347526.2_Capsaspora_owczarzaki
NP_001037626.1_Bombyx_mori
Nfowleri986_g3063.t1
NP_505629.2_Caenorhabditis_elegans
TTHERM_00310640_Tetrahymena_thermophila
Nfowleri986_g4272.t1
Nfowleri986_g10525.t1
Consensus
Conservation

Figure S4

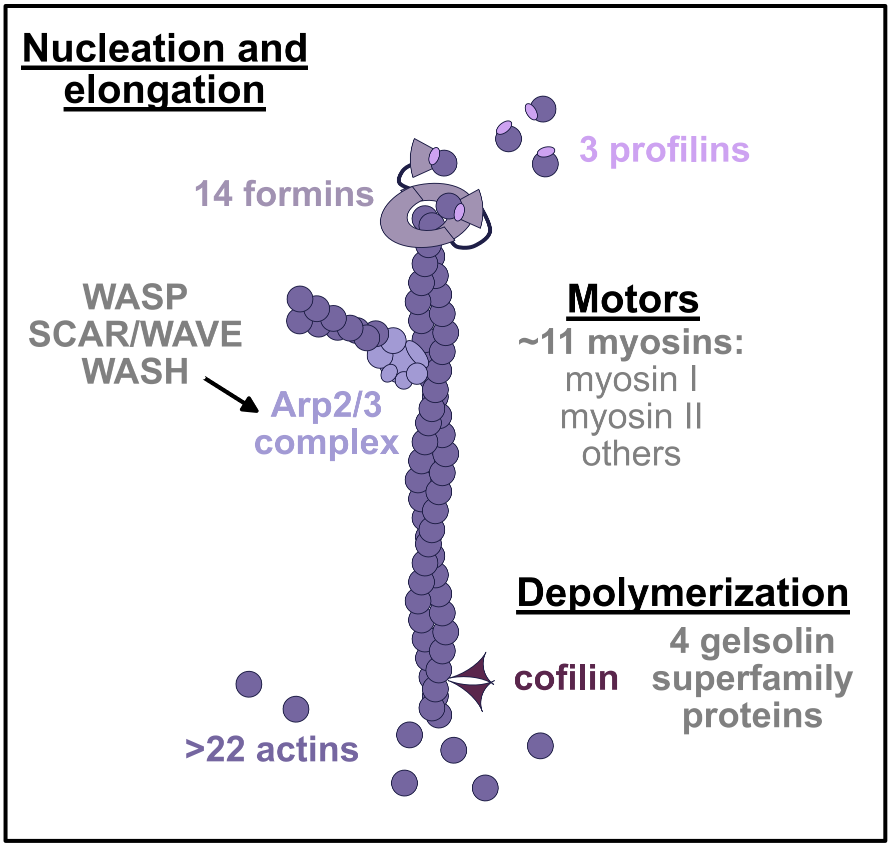
Nucleation and elongation
3 profilins
14 formins
WASP
SCAR/WAVE
WASH
Arp2/3 complex
Motors
~11 myosins:
myosin I
myosin II
others
Depolymerization
4 gelsolin superfamily proteins
cofilin
>22 actins

Figure S5

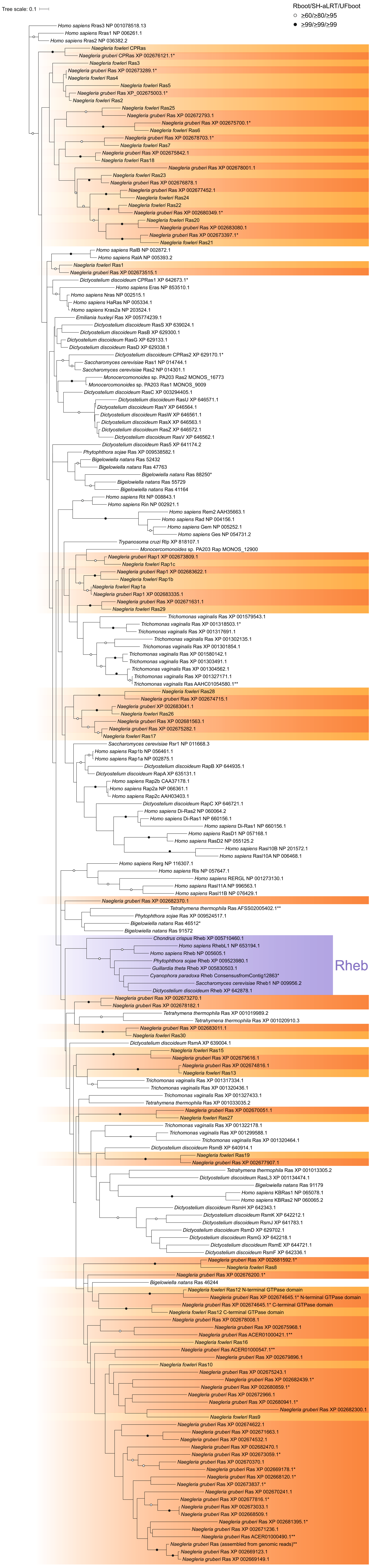
Tree scale: 0.1
Rboot/SH-aLRT/UFboot
≥60/≥80/≥95
≥99/≥99/≥99
Homo sapiens Rras3 NP 001078518.13
Homo sapiens Rras1 NP 006261.1
Homo sapiens Rras2 NP 036382.2
Naegleria fowleri CPRas
Naegleria gruberi CPRas XP 002676121.1*
Naegleria fowleri Ras3
Naegleria gruberi Ras XP 002673289.1*
Naegleria fowleri Ras4
Naegleria fowleri Ras5
Naegleria gruberi Ras XP_002675003.1*
Naegleria fowleri Ras2
Naegleria fowleri Ras25
Naegleria gruberi Ras XP 002672793.1
Naegleria gruberi Ras XP 002675700.1*
Naegleria fowleri Ras6
Naegleria gruberi Ras XP 002678703.1*
Naegleria fowleri Ras7
Naegleria gruberi Ras XP 002675842.1
Naegleria fowleri Ras18
Naegleria gruberi Ras XP 002678001.1
Naegleria fowleri Ras23
Naegleria gruberi Ras XP 002676878.1
Naegleria gruberi Ras XP 002677452.1
Naegleria fowleri Ras24
Naegleria fowleri Ras22
Naegleria gruberi Ras XP 002680349.1*
Naegleria fowleri Ras20
Naegleria gruberi Ras XP 002683080.1
Naegleria gruberi Ras XP 002673397.1*
Naegleria fowleri Ras21
Homo sapiens RalB NP 002872.1
Homo sapiens RalA NP 005393.2
Naegleria fowleri Ras1
Naegleria gruberi Ras XP 002673515.1
Dictyostelium discoideum CPRas1 XP 642673.1*
Homo sapiens Eras NP 853510.1
Homo sapiens Nras NP 002515.1
Homo sapiens HaRas NP 005334.1
Homo sapiens Kras2a NP 203524.1
Emiliania huxleyi Ras XP 005774239.1
Dictyostelium discoideum RasS XP 639024.1
Dictyostelium discoideum RasB XP 629300.1
Dictyostelium discoideum RasG XP 629133.1
Dictyostelium discoideum RasD XP 629338.1
Dictyostelium discoideum CPRas2 XP 629170.1*
Saccharomyces cerevisiae Ras1 NP 014744.1
Saccharomyces cerevisiae Ras2 NP 014301.1
Monocercomonoides sp. PA203 Ras2 MONOS_16773
Monocercomonoides sp. PA203 Ras1 MONOS_9009
Dictyostelium discoideum RasC XP 003294405.1
Dictyostelium discoideum RasU XP 646571.1
Dictyostelium discoideum RasY XP 646564.1
Dictyostelium discoideum RasW XP 646561.1
Dictyostelium discoideum RasX XP 646563.1
Dictyostelium discoideum RasZ XP 646572.1
Dictyostelium discoideum RasV XP 646562.1
Dictyostelium discoideum Ras5 XP 641174.2
Phytophthora sojae Ras XP 009538582.1
Bigelowiella natans Ras 52432
Bigelowiella natans Ras 47763
Bigelowiella natans Ras 88250*
Bigelowiella natans Ras 55729
Bigelowiella natans Ras 41164
Homo sapiens Rit NP 008843.1
Homo sapiens Rin NP 002921.1
Homo sapiens Rem2 AAH35663.1
Homo sapiens Rad NP 004156.1
Homo sapiens Gem NP 005252.1
Homo sapiens Ges NP 054731.2
Trypanosoma cruzi Rlp XP 818107.1
Monocercomonoides sp. PA203 Rap MONOS_12900
Naegleria gruberi Rap1 XP 002673809.1
Naegleria fowleri Rap1c
Naegleria gruberi Rap1 XP 002683622.1
Naegleria fowleri Rap1b
Naegleria fowleri Rap1a
Naegleria gruberi Rap1 XP 002683335.1
Naegleria gruberi Ras XP 002671631.1
Naegleria fowleri Ras29
Trichomonas vaginalis Ras XP 001579543.1
Trichomonas vaginalis Ras XP 001318503.1*
Trichomonas vaginalis Ras XP 001317691.1
Trichomonas vaginalis Ras XP 001302135.1
Trichomonas vaginalis Ras XP 001301854.1
Trichomonas vaginalis Ras XP 001580142.1
Trichomonas vaginalis Ras XP 001303491.1
Trichomonas vaginalis Ras XP 001304562.1
Trichomonas vaginalis Ras XP 001327171.1
Trichomonas vaginalis Ras AAHC01054580.1**
Naegleria fowleri Ras28
Naegleria gruberi Ras XP 002674715.1
Naegleria gruberi XP 002683041.1
Naegleria fowleri Ras26
Naegleria gruberi Ras XP 002681563.1
Naegleria gruberi Ras XP 002675282.1
Naegleria fowleri Ras17
Saccharomyces cerevisiae Rsr1 NP 011668.3
Homo sapiens Rap1b NP 056461.1
Homo sapiens Rap1a NP 002875.1
Dictyostelium discoideum RapB XP 644935.1
Dictyostelium discoideum RapA XP 635131.1
Homo sapiens Rap2b CAA37178.1
Homo sapiens Rap2a NP 066361.1
Homo sapiens Rap2c AAH03403.1
Dictyostelium discoideum RapC XP 646721.1
Homo sapiens Di-Ras2 NP 060064.2
Homo sapiens Di-Ras1 NP 660156.1
Homo sapiens Di-Ras1 NP 660156.1
Homo sapiens RasD1 NP 057168.1
Homo sapiens RasD2 NP 055125.2
Homo sapiens Rasl10B NP 201572.1
Homo sapiens Rasl10A NP 006468.1
Homo sapiens Rerg NP 116307.1
Homo sapiens Ris NP 057647.1
Homo sapiens RERGL NP 001273130.1
Homo sapiens Rasl11A NP 996563.1
Homo sapiens Rasl11B NP 076429.1
Naegleria gruberi Ras XP 002682370.1
Tetrahymena thermophila Ras AFSS02005402.1**
Phytophthora sojae Ras XP 009524517.1
Bigelowiella natans Ras 46512*
Bigelowiella natans Ras 91572
Chondrus crispus Rheb XP 005710460.1
Homo sapiens RhebL1 NP 653194.1
Homo sapiens Rheb NP 005605.1
Phytophthora sojae Rheb XP 009523980.1
Guillardia theta Rheb XP 005830503.1
Cyanophora paradoxa Rheb ConsensusfromContig12863*
Saccharomyces cerevisiae Rheb1 NP 009956.2
Dictyostelium discoideum Rheb XP 642878.1
Rheb
Naegleria gruberi Ras XP 002673270.1
Naegleria gruberi Ras XP 002678182.1
Tetrahymena thermophila Ras XP 001019989.2
Tetrahymena thermophila Ras XP 001020910.3
Naegleria gruberi Ras XP 002683011.1
Naegleria fowleri Ras30
Dictyostelium discoideum RsmA XP 639004.1
Naegleria fowleri Ras15
Naegleria gruberi Ras XP 002679616.1
Naegleria gruberi Ras XP 002674816.1
Naegleria fowleri Ras13
Trichomonas vaginalis Ras XP 001317334.1
Trichomonas vaginalis Ras XP 001320436.1
Trichomonas vaginalis Ras XP 001327433.1
Tetrahymena thermophila Ras XP 001033035.2
Naegleria gruberi Ras XP 002670051.1
Naegleria fowleri Ras27
Trichomonas vaginalis Ras XP 001322178.1
Trichomonas vaginalis Ras XP 001299588.1
Trichomonas vaginalis Ras XP 001320464.1
Dictyostelium discoideum RsmB XP 640914.1
Naegleria fowleri Ras19
Naegleria gruberi Ras XP 002677907.1
Tetrahymena thermophila Ras XP 001013305.2
Dictyostelium discoideum RasL3 XP 001134474.1
Bigelowiella natans Ras 91179
Homo sapiens KBRas1 NP 065078.1
Homo sapiens KBRas2 NP 060065.2
Dictyostelium discoideum RsmH XP 642343.1
Dictyostelium discoideum RsmK XP 642212.1
Dictyostelium discoideum RsmJ XP 641783.1
Dictyostelium discoideum RsmD XP 629702.1
Dictyostelium discoideum RsmG XP 642218.1
Dictyostelium discoideum RsmE XP 644721.1
Dictyostelium discoideum RsmF XP 642336.1
Naegleria gruberi Ras XP 002681592.1*
Naegleria fowleri Ras8
Naegleria gruberi Ras XP 002676200.1*
Bigelowiella natans Ras 46244
Naegleria fowleri Ras12 N-terminal GTPase domain
Naegleria gruberi Ras XP 002674645.1* N-terminal GTPase domain
Naegleria gruberi Ras XP 002674645.1* C-terminal GTPase domain
Naegleria fowleri Ras12 C-terminal GTPase domain
Naegleria gruberi Ras XP 002678008.1
Naegleria gruberi Ras XP 002675968.1
Naegleria gruberi Ras ACER01000421.1**
Naegleria fowleri Ras16
Naegleria gruberi Ras ACER01000547.1**
Naegleria gruberi Ras XP 002679896.1
Naegleria fowleri Ras10
Naegleria gruberi Ras XP 002675243.1
Naegleria gruberi Ras XP 002682439.1*
Naegleria gruberi Ras XP 002680859.1*
Naegleria gruberi Ras XP 002672966.1
Naegleria gruberi Ras XP 002680941.1*
Naegleria gruberi Ras XP 002682300.1
Naegleria fowleri Ras9
Naegleria gruberi Ras XP 002674622.1
Naegleria gruberi Ras XP 002671663.1
Naegleria gruberi Ras XP 002674532.1
Naegleria gruberi Ras XP 002682470.1
Naegleria gruberi Ras XP 002673059.1*
Naegleria gruberi Ras XP 002670370.1
Naegleria gruberi Ras XP 002669178.1*
Naegleria gruberi Ras XP 002668120.1*
Naegleria gruberi Ras XP 002673837.1*
Naegleria gruberi Ras XP 002670241.1
Naegleria gruberi Ras XP 002677816.1*
Naegleria gruberi Ras XP 002673033.1
Naegleria gruberi Ras XP 002668509.1
Naegleria gruberi Ras XP 002681395.1*
Naegleria gruberi Ras XP 002671236.1
Naegleria gruberi Ras ACER01000490.1**
Naegleria gruberi Ras (assembled from genomic reads)**
Naegleria gruberi Ras XP 002669123.1
Naegleria gruberi Ras XP 002669149.1

Figure S6

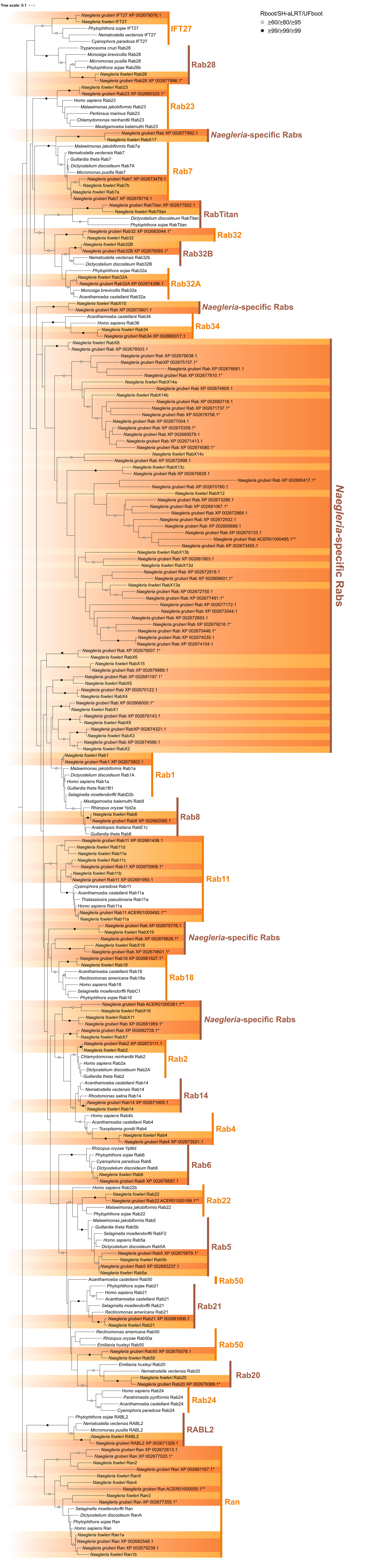
Tree scale: 0.1
Rboot/SH-aLRT/UFboot
≥60/≥80/≥95
≥99/≥99/≥99
IFT27
Rab28
Rab23
Naegleria-specific Rabs
Rab7
RabTitan
Rab32
Rab32B
Rab32A
Naegleria-specific Rabs
Rab34
Naegleria-specific Rabs
Rab1
Rab8
Rab11
Naegleria-specific Rabs
Rab18
Naegleria-specific Rabs
Rab2
Rab14
Rab4
Rab6
Rab22
Rab5
Rab50
Rab21
Rab50
Rab20
Rab24
RABL2
Ran

Figure S7

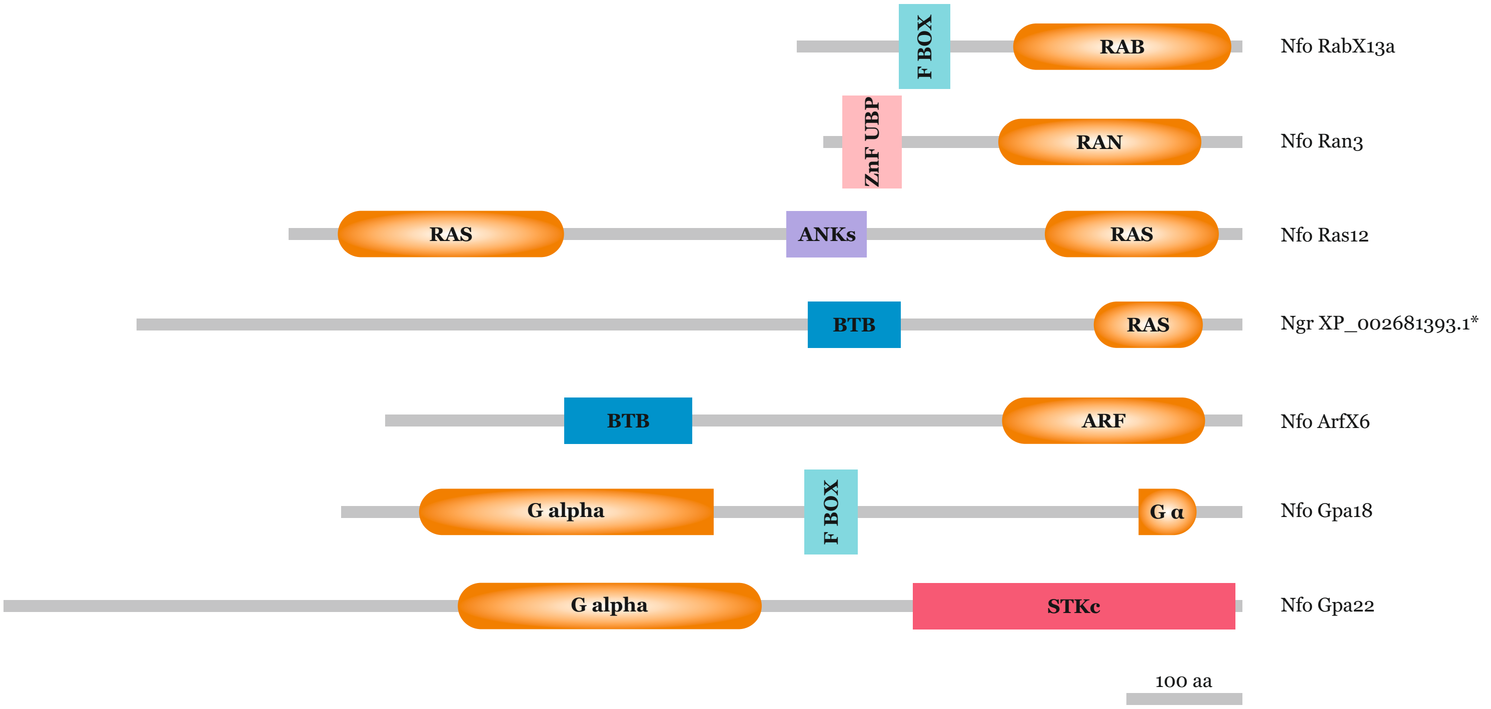
F BOX
RAB
Nfo RabX13a
ZnF UBP
RAN
Nfo Ran3
RAS
ANKs
RAS
Nfo Ras12
BTB
RAS
Ngr XP_002681393.1*
BTB
ARF
Nfo ArfX6
G alpha
F BOX
G α
Nfo Gpa18
G alpha
STKc
Nfo Gpa22
100 aa

Figure S8

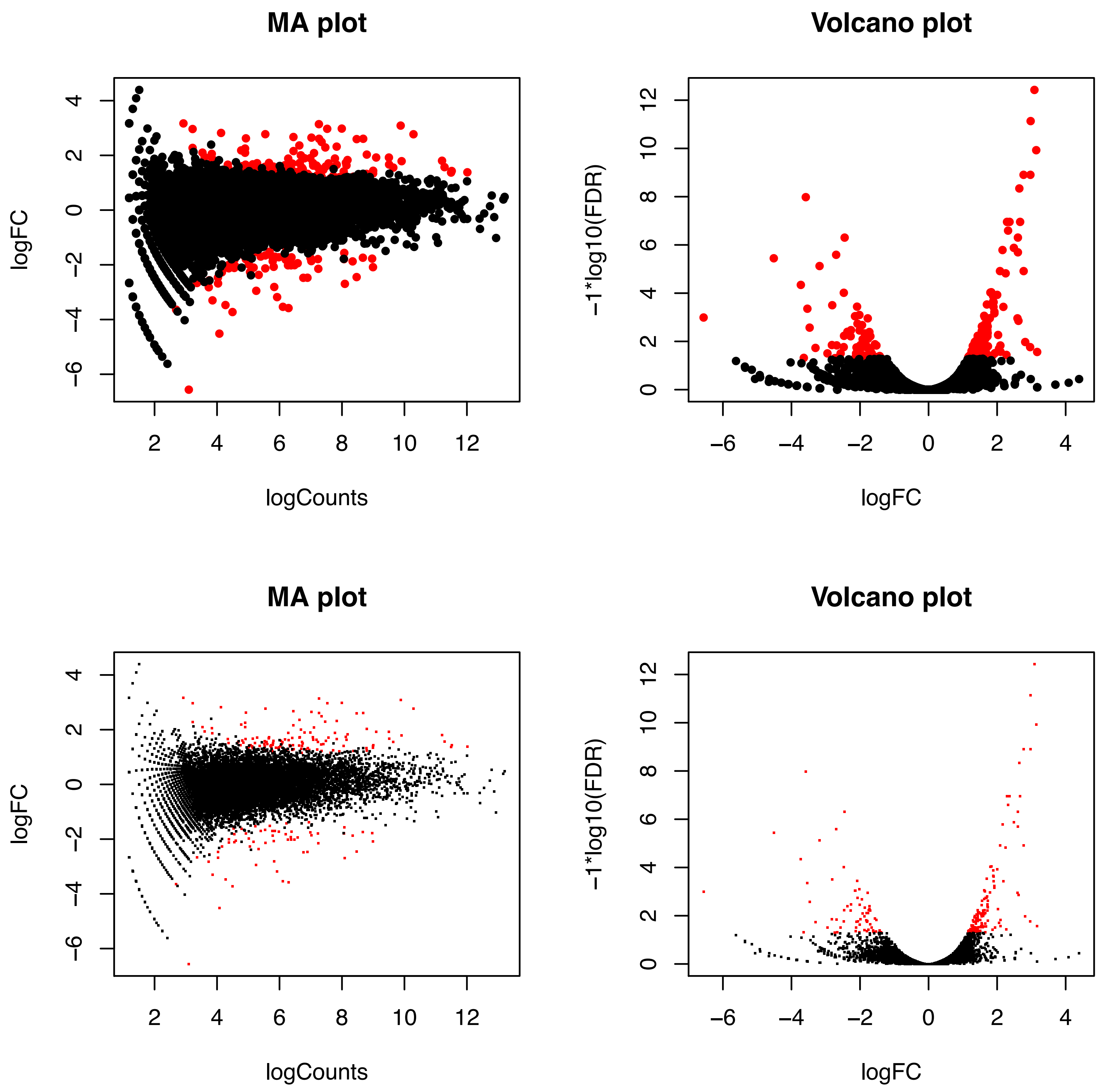
MA plot
Volcano plot
logFC
logCounts
−1*log10(FDR)
logFC
MA plot
Volcano plot
logFC
logCounts
−1*log10(FDR)
logFC

Figure S9

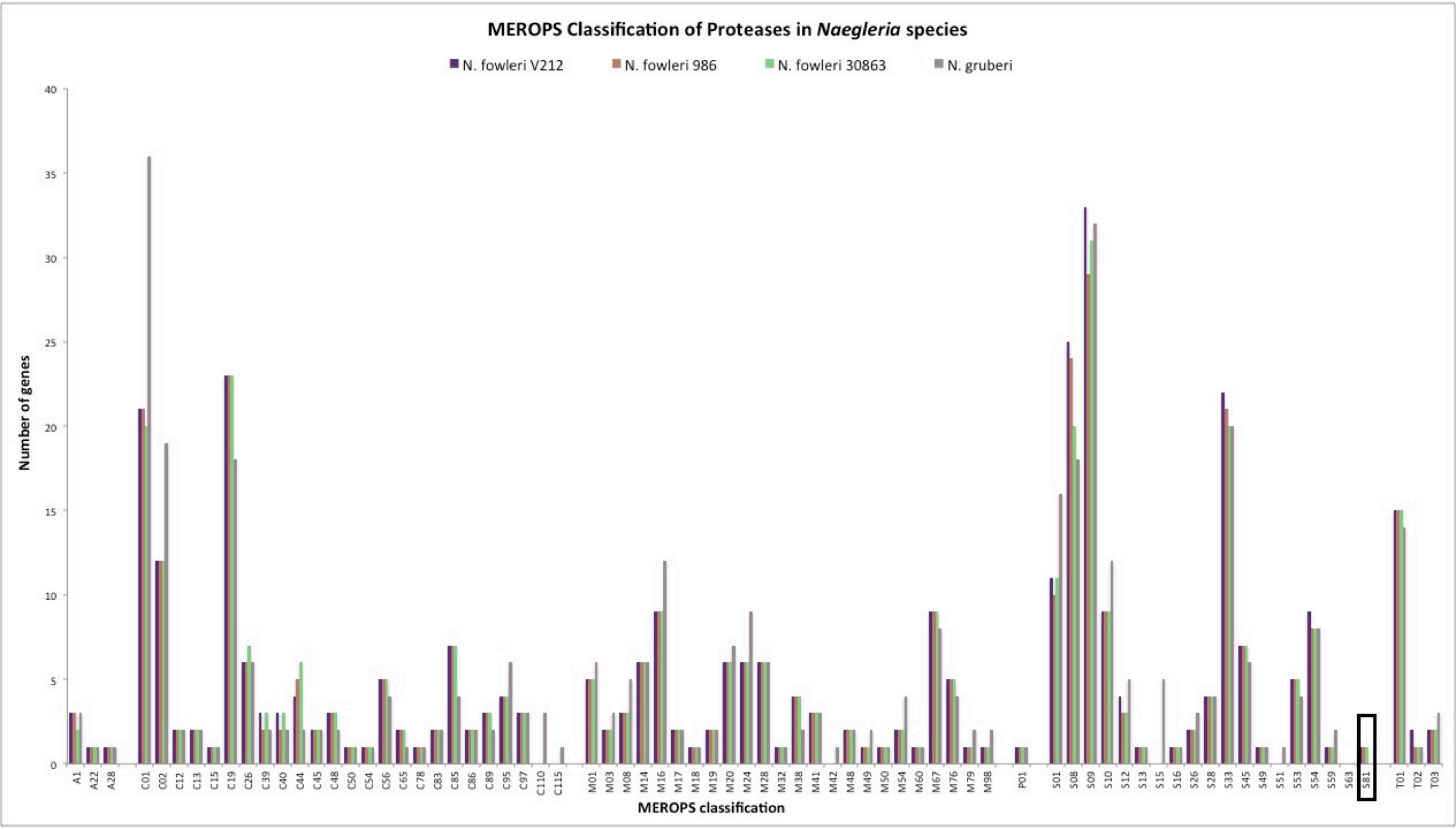
MEROPS Classification of Proteases in *Naegleria* species
N. fowleri V212
N. fowleri 986
N. fowleri 30863
N. gruberi
Number of genes
0
5
10
15
20
25
30
35
40
A1
A22
A28
C01
C02
C12
C13
C15
C19
C26
C39
C40
C44
C45
C48
C50
C54
C56
C65
C78
C83
C85
C86
C89
C95
C97
C110
C115
M01
M03
M08
M14
M16
M17
M18
M19
M20
M24
M28
M32
M38
M41
M42
M48
M49
M50
M54
M60
M67
M76
M79
M98
P01
S01
S08
S09
S10
S12
S13
S15
S16
S26
S28
S33
S45
S49
S51
S53
S54
S59
S63
S81
T01
T02
T03
MEROPS classification

Figure S10

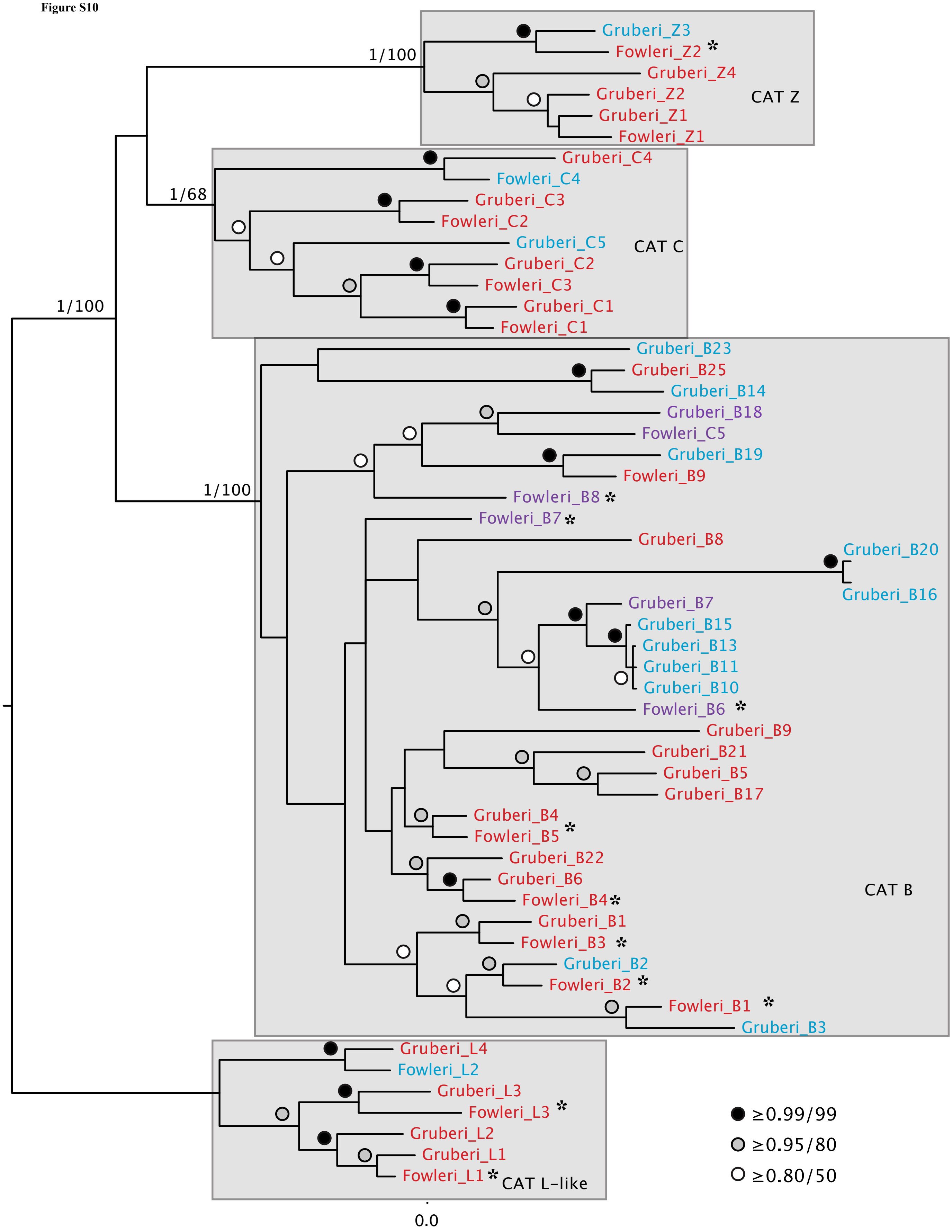
1/100
Gruberi_Z3
Fowleri_Z2
Gruberi_Z4
Gruberi_Z2
Gruberi_Z1
Fowleri_Z1
CAT Z
1/68
Gruberi_C4
Fowleri_C4
Gruberi_C3
Fowleri_C2
Gruberi_C5
Gruberi_C2
Fowleri_C3
Gruberi_C1
Fowleri_C1
CAT C
1/100
1/100
Gruberi_B23
Gruberi_B25
Gruberi_B14
Gruberi_B18
Fowleri_C5
Gruberi_B19
Fowleri_B9
Fowleri_B8
Fowleri_B7
Gruberi_B8
Gruberi_B20
Gruberi_B16
Gruberi_B7
Gruberi_B15
Gruberi_B13
Gruberi_B11
Gruberi_B10
Fowleri_B6
Gruberi_B9
Gruberi_B21
Gruberi_B5
Gruberi_B17
Gruberi_B4
Fowleri_B5
Gruberi_B22
Gruberi_B6
Fowleri_B4
Gruberi_B1
Fowleri_B3
Gruberi_B2
Fowleri_B2
Fowleri_B1
Gruberi_B3
CAT B
Gruberi_L4
Fowleri_L2
Gruberi_L3
Fowleri_L3
Gruberi_L2
Gruberi_L1
Fowleri_L1
CAT L-like
≥0.99/99
≥0.95/80
≥0.80/50
0.0
