# Supplementary material for: Genomics and transcriptomics yields a system-level view of the biology of the pathogen Naegleria fowleri
Source: BMC Biol. 2021 Jul 22;19:142. doi: 10.1186/s12915-021-01078-1 (PMC8296547; doi:10.1186/s12915-021-01078-1)
Supplement: Supplementary file 4 — Additional file 4: Supplementary Material 1. Transcription factor identification. Supplementary Material 2. Sterol metabolism genes from Naegleria fowleri. Supplementary Material 3. Analysis of the cytoskeletal protein complement in N. fowleri. Supplementary Material 4. Ras superfamily GTPases in Naegleria spp. [file 12915_2021_1078_MOESM4_ESM.docx]

**Supplementary Material 1: Transcription factor identification**

We identified likely transcription factors (TFs) by scanning the amino acid sequences of predicted protein coding genes for putative DNA binding domains (DBDs) using the procedures described in Weirauch et al 2014 [32]*.* Briefly, we scanned all protein sequences for putative DBDs using the 81 Pfam [34] models listed in Weirauch and Hughes 2011 [33]and the HMMER tool [35], with the recommended detection thresholds of Per-sequence Eval < 0.01 and Per-domain conditional Eval < 0.01. Each protein was classified into a family based on its DBDs and their order in the protein sequence (e.g., bZIPx1, AP2x2, Homeodomain+Pou). Using the above procedure, we identified a total of 203, 196, and 196 putative TFs in the genomes of *N. fowleri* strains 30863, 986, and V212, respectively. These values are similar to those reported previously for *N. gruberi* (243), especially when accounting for the fact that *N. gruberi* has ~30% more genes than *N. fowleri*. A few TF families are under-represented in *N. fowleri* relative to *N. gruberi* (e.g., zinc clusters, AT hooks, and HMG boxes), and one family (GATA) is substantially expanded (10 vs 4 genes).

**Supplementary Material 2: Sterol metabolism genes from *Naegleria fowleri***

Sterols are fundamental lipids in eukaryotes. They are important components of the plasma membrane, playing relevant structural roles and also participating in cellular signaling. Due to its essentiality and prevalence in eukaryotic microorganisms[36], sterol metabolism is a frequent therapeutic target against pathogenic fungi and microbial parasites including kinetoplastids and various amoebae [37].

A sterol biosynthesis pathway was proposed two decades ago in the non-pathogenic *Naegleria gruberi* and *N. lovaniensis* [39]. Like fungi and kinetoplastids, *Naegleria* species use ergosterol as the major sterol component of their membranes. While fungi and kinetoplastids use lanosterol as a starting point, *Naegleria* species produce ergosterol through cycloartenol as the first cyclic product of the pathway (a characteristic shared with some photosynthetic lineages). Because efforts in developing effective treatments for *N. fowleri* infections rely on repurposing of existing drugs (e.g. antifungals), it is imperative to determine the identity of the enzymatic steps involved in the biosynthesis of ergosterol in *N. fowleri* [40].

The putative sterol biosynthesis pathway of *N. fowleri* was reconstructed using BLASTp to mine the predicted proteomes from the three strains studied here. Amino acid sequences of sterol metabolism proteins from *Homo sapiens*, *Saccharomyces cerevisiae* and *Arabidopsis thaliana*, were used as queries. We have also searched for orthologues of the cholesterol C7(8)-desaturase (EC 1.14.19.21).

We have found the complete set of proteins for the production of sterols in the three *N. fowleri* strains investigated (Additional File 3- Figure S2). We have also detected putative orthologues of the Rieske cholesterol C7(8)-desaturase (Additional File 2- Figure S2). This enzyme converts cholesterol into 7-dehydrocholesterol during steroid hormones synthesis in ecdysozoans [41-42], in the ciliate *Tetrahymena thermophila* [43], and was also proposed to be involved in the pathway of conversion of diet cholesterol into ergosterol in the unicellular amoeba *Capsaspora owczarzaki* [44]. Importantly, cholesterol 7(8)-desaturase is highly conserved in animals except mammals [43], thus providing a potential candidate target to design drugs to abolish or restrict the synthesis of sterols in *N. fowleri* without affecting the human host.

Like in *N. gruberi*, there are no orthologs of sterol C7-reductase in *N. fowleri*. This is not surprising if we assume that the pathway will lead to ergosterol as the end product, like in the non-pathogenic *Naegleria* species. Also, we were unable to detect an ortholog of ERG5/CYP710A1, the cytochrome P450 C22-desaturase. Desaturation at position C22(23) of the lateral chain is necessary for the production of ergosterol. However, the absence of an ERG5/CYP701A1 ortholog does not necessarily mean that *Naegleria* *spp*. are not capable of performing this enzymatic activity. The choanoflagellate *Monosiga brevicolis* [45] and the ciliate *Tetrahymena thermophila* [46] are examples of species known to produce delta-22-sterols, without having a canonical P450 sterol C22-desaturase [38].

None of the genes encoding sterol metabolism enzymes exhibited significant changes in transcript levels in our RNAseq experiments. The only gene related to this metabolic pathway that is differentially expressed is the squalene synthase (SQS). However, squalene is the building block of many isoprenoids, not only sterols. Thus, a direct link between the overexpression of SQS and the overproduction of sterols (and hence its involvement in the infection process) cannot be suggested.

**Supplementary Material 3: Analysis of the cytoskeletal protein complement in *N. fowleri***

The actin and microtubule cytoskeletons coordinate and execute nearly every cellular function, including migration, endocytosis, and cell division. In addition to clear roles in cell growth and viability, these functions likely drive pathogenesis (reviewed in [59]); after inhalation of contaminated water, *N. fowleri* migrates to and within the brain [60], where endocytosis of host material [61-62] and release of mucus- and tissue-degrading enzymes cause damage and inflammation [21, 24, 63]. To understand the mechanisms underlying these pathogenic behaviours, we need a clear picture of the composition of the microtubule and actin cytoskeletons. Much of what we know about *Naegleria*’s cytoskeleton pertains to its unique differentiation from amoebae into flagellates. During this transition, *Naegleria* synthesize all the proteins required to build flagella, including basal bodies and flagellar tubulin [27,47-48,64]. In addition, *Naegleria* presumably uses microtubules for chromosome segregation during closed mitosis [49-50]. We find that *N. fowleri* and the non-pathogenic species *Naegleria gruberi* each possess an extensive repertoire of tubulins and microtubule associated proteins (Additional File 8 Table S6). We identified over 140 genes encoding proteins involved in the microtubule cytoskeleton in *N. fowleri*, including; several tubulins (including >13 alpha and beta tubulins, gamma, delta, and epsilon tubulins), motor proteins (>30 kinesins and 6 dyneins (based on the number of heavy chains)), as well as proteins associated with interflagellar transport, basal body assembly and structure [27,64], and many other microtubule binding proteins. Despite this extensive microtubule gene repertoire, infectious *Naegleria* amoebae move, eat, and divide without cytoplasmic microtubules [49, 60] Moreover, previous studies have suggested that actin, actin binding proteins, or upstream actin regulators may correlate with virulence [28, 51]. Therefore, a thorough understanding of *Naegleria*’s control of actin dynamics remains critical to understanding its pathogenesis. In cells, actin polymer formation requires proteins called nucleators [52-53]. The Arp2/3 complex is a nucleator that typically generates branched actin networks that are useful for cell motility and vesicle trafficking [52, 54]. We predict that *N. fowleri* can form these branched actin networks, as we identified all seven subunits of the Arp2/3 complex (Additional File 8- Table S6). We also found upstream Arp2/3 activators from the WASP-family, including WASP and all components of the WASH and SCAR/WAVE complexes. The presence of WASP and SCAR/WAVE together is indicative of the capacity for pseudopod-based alpha-motility [55], which is consistent with microscopic observations [56-57]. In addition to Arp2/3 complex-mediated nucleation, cells also use formin family proteins to nucleate and elongate actin polymers (Additional File 3-Figure S4). Formin proteins have highly variable domain architectures, and can also bundle and sever actin filaments [58]. We identified 14 formin homology 2 (FH2) domain-containing proteins, and of those 12 have similar domains and organization to diaphanous related formins (DRFs), and two contain phosphatase and tensin (PTEN) domains. Because amoebae rely on actin for their cytoskeleton, and because actin regulation often occurs at the nucleation level, these Arp2/3- and formin-mediated pathways of actin polymerization likely drive most cellular functions. As an alternative strategy to identify actin related pathogenicity factors, we compared *N. fowleri* cytoskeletal genes to those of its non-pathogenic relative, *N. gruberi* (Additional File 8- Table S6). Generally, *N. fowleri* and *N. gruberi* harbour similar numbers of the same genes. Because actin is regulated largely at the level of nucleation, we classified the *N. gruberi* and *N. fowleri* formin families by domain composition and found a single difference in one formin conserved in the two species. In this formin (62754), the *N. fowleri* homolog has a putative lipid-binding PTEN domain that is missing from that of *N. gruberi*. While the comparison between *Naegleria* strains did not reveal any major differences in the cytoskeletal repertoires that can immediately explain the differences in pathogenicity, it is notable that humans do not encode formins of the PTEN family. Therefore, if these PTEN formins are responsible for any vital *Naegleria* processes, they may represent useful drug targets. Because the cytoskeleton is central to *N. fowleri* viability and pathogenesis, and because actin-based processes likely dictate pathogenic behaviours, these analyses provide a solid framework for future investigation into *N. fowleri* virulence as well as potential drug targets.

**Supplementary Material 4: Ras superfamily GTPases in *Naegleria* spp.**

The Ras superfamily of GTPases represents a vast group of proteins involved in endomembrane transport, cell signalling, actin dynamics, cilium-associated functions, and many other eukaryote-specific processes [65]. The previously published *Naegleria gruberi* genome sequence revealed a plethora of Ras superfamily members, including “monomeric Ras-like GTPases” (182 genes identified by the Pfam profile PF00071) and alpha subunits of heterotrimeric G proteins (Gα; 39 genes identified by the Pfam profile PF00503) [27]. In order to assess the differences between *N. gruberi* and *N. fowleri*, we annotated the three strains of *N. fowleri* and also carefully reannotated the complement of Ras superfamily genes in the *N. gruberi* genome.

Reannotation of the complement of Ras superfamily genes in *N. gruberi* yielded over 350 Ras superfamily genes. Compared to the original annotation [27], many incorrectly predicted gene models were fixed and a number of genes that had escaped annotation were identified and annotated anew (the reannotated set of the *N. gruberi* Ras superfamily GTPases is listed in Additional File 10-Table S8). The Ras superfamily complement in *N. fowleri* is much less expanded, amounting to “only” over 200 genes (all listed in Additional File 10-Table S8, sheet 2), which is nevertheless still a lot compared to the numbers usually seen in eukaryotes. Little, if any, differences were found between the three *N. fowleri* strains (Additional File 10-Table S8, sheet 2). A substantial part (>180) of the genes exhibits an obvious one-to-one orthology relationship between *N. gruberi* and *N. fowleri* (Additional File 10-Table S8, sheet 2), but paralog expansions have extensively shaped the complements of Ras superfamily genes after the divergence of the lineages leading these two species. However, *N. gruberi* and much less frequently *N. fowleri* exhibit Ras superfamily genes lacking obvious orthologs in the other species. These may represent extremely divergent lineage-specific paralogs or genes predating the two species but lost in one or the other species. Genome analyses of other heteroloboseans are required to distinguish between these two possibilities.

Our analyses revealed that *Naegleria* inherited a large complement of Ras superfamily genes form an ancestor shared with other eukaryotes: both *Naegleria* spp. exhibit apparent orthologs of more than 50 Ras superfamily members from eukaryotes outside Heterolobosea (Additional File 10-Table S8, sheet 2; note that eukaryote-wide phylogenetic history of some subgroups of the Ras superfamily, e.g. the Gα proteins, has not been extensively investigated yet, so our result concerning orthologous relationships between *Naegleria* and other eukaryotes is a minimal estimate). There are few known conserved Ras superfamily members common in other eukaryotes yet missing in *Naegleria*, notable examples being Rab24 [66 or proteins of the Roco family [67]. It was previously reported that *N. gruberi* has the GTPase Rheb [68], but the respective gene (XP_002683011.1) and its *N. fowleri* ortholog (Ras30) are more likely divergent paralogs of the true Ras (Additional File 3-Figure S5), whereas the *bona fide* Rheb was lost in the *Naegleria* lineage, yet retained by some other heteroloboseans [67].

Interesting commonalities as well as differences are seen when individual main subgroups of the Ras superfamily are analysed in the two *Naegleria* species. 44 Rab and Rab-like genes were previously reported from *N. gruberi* [66]. We now additionally identified a number of more divergent Rab-like genes in the *N. gruberi* genome, expanding the size of the Rab(-like) family to nearly 90 loci in this species, whereas the equivalent group of genes comprises at least 55 Rab-like loci in *N. fowleri*. The difference stems essentially only from differential expansion of three subgroups of divergent Rab-like genes lacking discernible close relatives in eukaryotes outside *Naegleria* (Additional File 3-Figure S5). Interestingly, some members of the three divergent Rab-like clades in both *Naegleria* species seem to be affected by mutations disrupting the coding sequence as predicted based on conservation with the related genes, suggesting a recent dynamic birth-and-death evolution. Proteins in each of the three subgroups possess a conserved N-terminal extension upstream of the GTPase domain, in at least some of these proteins identifiable as the F-box domain (see also Additional File 3-Figure S7). One of these three subgroups has a conserved C-terminal prenylation motif (in the CaaX form; [70]), suggesting that it is geranylgeranylated (or farnesylated) and attaches to membranes like standard Rabs and some other Ras superfamily GTPases. No specific functional prediction for these proteins is possible from the sequence analysis only, but their involvement in processes at the organism-environment interface seems likely.

We detected 80 and more than 40 genes representing the Ras family in *N. gruberi* and *N. fowleri*, respectively. These include the previously detected CPRas protein characterized by a unique circularly-permuted GTPase domain [71] and three paralogs of the GTPase Rap1 (Additional File 3-Figure S5). The remaining Ras family genes in *Naegleria* species all seem to derive from the ancestral true Ras protein [72] based on sequence similarity comparisons, although many of the paralogs in *Naegleria* spp. are too divergent to prove this by phylogenetic analyses. The Rho family is represented by nearly 50 and more than 80 genes in *N. fowleri* and *N. gruberi*, respectively. Interestingly, the family also includes the RhoBTB type (proteins with a Rho-like GTPase domain fused to a tandem of BTB domains) so far known only from metazoans and the amoebozoan *Dictysotelium* [73]. Expanded families of Ran GTPase homologs are rarely seen in eukaryotes, so it is interesting to note that *N. fowleri* and *N. gruberi* harbor six and seven Ran or Ran-like genes, respectively (including one presumable pseudogene in the former, Additional File 10-Table S8). These include not only paralogs highly similar to canonical Ran from other eukaryotes and thus apparently mediating nucleocytoplasmic transport [74], but also several unusual more divergent paralogs, some of them provided with N-terminal extra domains (see below and Additional File 3-Figure S7), suggesting their possible recruitment for novel cellular roles. The Arf/Arl/Sar1 family is not particularly expanded in *Naegleria*, but the two species analysed do differ in a complement of several divergent taxon-specific paralogs of unknown function. Finally, our reanalysis of the *N. gruberi* genome revealed that the Gα family (alpha subunits of heterotrimeric G-proteins) is even larger than reported previously (39 genes identified by [23]). We now detected 60 Gα genes in this species, whereas *N. fowleri* has a smaller, but still unusually large complement of >30 Gα genes.

The GTPase domain of the Ras superfamily typically exists as a stand-alone protein (i.e. a “small GTPase”), possibly with an (often prenylated or acylated) N- or C-terminal extension mediating membrane attachment, but it can be also incorporated into larger proteins featuring additional functional domains. In addition to several multi-domain Ras superfamily proteins representing ancestral or at least widespread forms, e.g. CPRas, RhoBTB, Miro [71, 73, 75], both *Naegleria* spp. exhibit a number of genes with novel domain architectures including a Ras superfamily GTPase domain as one of the elements (listed in Additional File S10-Table S8, examples provided in Additional File 3-Figure S7). The most frequent additional domain is the F-box found in combination with GTPase domains belonging to the Rab, Rho, Ran, and Gα families. Curiously, in the latter case the F-box domain is nested within the GTPase domain. We also identified one Ras protein and several Arf and Arf-like proteins combined with the BTB domain. Both F-box and BTB domains are implicated in protein-protein interactions, particularly in the context of protein ubiquitination as components of ubiquitin ligases [76-77], pointing to GTPase-dependent ubiquitination as a possibly significant way of cellular regulation in *Naegleria*. In addition to domains mentioned above, we also identified combinations of a Ras superfamily GTPase domain with, e.g., a serine/threonine protein kinase domain, LRR repeats, Ankyrin repeats, or Kelch motifs.

Our analyses revealed that amoeboflagellates of the genus *Naegleria* possess a highly complex and evolutionarily dynamic set of Ras superfamily genes that points to a hidden level of differentiation in cellular physiology of different *Naegleria* species. Interestingly, a similar pattern is seen in members of Amoebozoa (see, e.g., [78]), so we speculate that an amoeboid lifestyle generally requires a complex GTPase-based regulation of cellular activities. For example, in analogy to results obtained by studying metazoan and *Dictyostelium* amoeboid cells [79], proteins from the highly expanded Ras, Rho, and Gα families may underpin sophisticated functional networks responsible for sensing and reacting to various chemoattractants. Unfortunately, testing this hypothesis is precluded by the current lack of tools for genetic manipulation of *Naegleria* spp.
